# Liquid-Phase Exfoliation of Arsenic Trisulfide (As_2_S_3_) Nanosheets and Their Use as Anodes in Potassium-Ion Batteries

**DOI:** 10.1021/acsnano.4c03501

**Published:** 2024-07-22

**Authors:** Harneet Kaur, Bharathi Konkena, Mark McCrystall, Kevin Synnatschke, Cian Gabbett, Jose Munuera, Ross Smith, Yumei Jiang, Raman Bekarevich, Lewys Jones, Valeria Nicolosi, Jonathan N Coleman

**Affiliations:** † School of Physics, CRANN & AMBER Research Centres, Trinity College Dublin, Dublin 2 D02 E8C0, Ireland; ‡ School of Chemistry, CRANN & AMBER Research Centres, Trinity College Dublin, Dublin 2 D02W9K7, Ireland

**Keywords:** two-dimensional materials, arsenic disulfide, liquid-phase exfoliation, nanosheets, K-ion battery anodes, rate performance

## Abstract

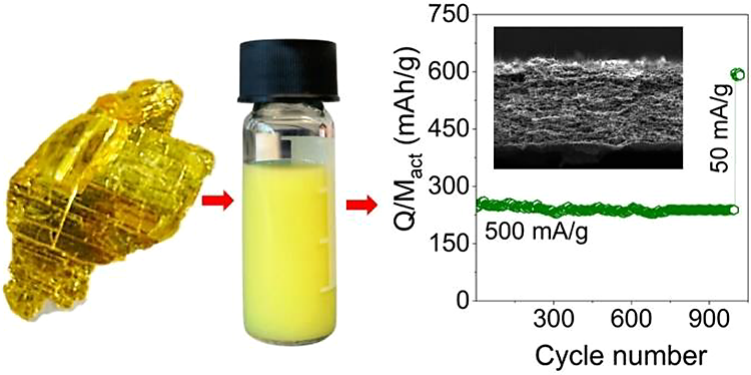

Here, we demonstrate the production of 2D nanosheets of arsenic disulfide (As_2_S_3_) via liquid-phase exfoliation of the naturally occurring mineral, orpiment. The resultant nanosheets had mean lateral dimensions and thicknesses of 400 and 10 nm, and had structures indistinguishable from the bulk. The nanosheets were solution mixed with carbon nanotubes and cast into nanocomposite films for use as anodes in potassium-ion batteries. These anodes exhibited outstanding electrochemical performance, demonstrating an impressive discharge capacity of 619 mAh/g at a current density of 50 mA/g. Even after 1000 cycles at 500 mA/g, the anodes retained an impressive 94% of their capacity. Quantitative analysis of the rate performance yielded a capacity at a very low rate of 838 mAh/g, about two-thirds of the theoretical capacity of As_2_S_3_ (1305 mAh/g). However, this analysis also implied As_2_S_3_ to have a very small solid-state diffusion coefficient (∼10^–17^ m^2^/s), somewhat limiting its potential for high-rate applications.

## Introduction

The escalating global demand for energy storage solutions, driven by the widespread popularity of portable electronic devices,^1^ the rise of electric vehicles,^2^ and the integration of renewable energy sources,^3^ has contributed to the development of lithium-ion batteries (LIBs) as today's predominant energy storage technology.^3,4^ However, the relentless expansion of battery usage has highlighted the limited global supply of lithium.^5^ In turn, this has raised concerns regarding the vulnerability of our dependence on lithium, prompting a re-evaluation of our energy storage approaches.^5,6^ In this context, the spotlight has turned to developing viable alternatives to LIBs. In particular, much attention has been given to batteries based on sodium and potassium ions due to their chemical similarity to lithium.^7−9^ Compared to lithium, sodium and potassium are abundant elements and their use in batteries would enhance sustainability and accessibility.^9,10^

While substantial progress has been achieved in the domain of sodium-ion batteries (SIBs),^11,12^ the development of potassium-ion batteries (KIBs) is less mature.^8,13,14^ There is still a pressing need to develop and characterize new materials for use as both cathodes^15^ and anodes^16^ in KIBs. For over a decade, the broad family of two-dimensional (2D) layered materials has shown immense promise for use in both LIB and SIB electrodes.^17,18^ More recently, these materials have been utilized in the pursuit of new potassium-storing anode materials.^19^ The appeal of 2D materials for use in anodes derives from various attributes. For example, the family of 2D materials is very diverse, containing >2000 known materials, each with distinct properties and capabilities.^20^ This huge material choice increases the probability of discovering a material with the right combination of properties to meet the specific demands of KIBs. In addition, the ability to convert layered materials into nanosheets via liquid exfoliation^21,22^ and subsequently to control their dimensions via size-selection protocols^23^ enables a high degree of processability, allowing us to produce electrodes with controlled properties.^24,25^ Finally, we might expect diffusion of ions within the interlayer channels in 2D materials to be faster than in 3D materials, leading to better rate performance and enhanced power density.^24−26^

Thus, while 2D materials are very attractive for use in KIBs, only a fraction of the vast array of possible 2D materials has been explored.^19^ The quest for high-capacity electrodes had led to a focus on phosphorus- or sulfur-rich materials such as black phosphorus (b-P, theoretical capacity = 864 mAh/g^27^), tin sulfide (SnS_2_, 733 mAh/g^28^), molybdenum disulfide (MoS_2_, 670 mAh/g^29,30^), antimony trisulfide (Sb_2_S_3_, 946 mAh/g^31,32^), or bismuth trisulfide (Bi_2_S_3_, 625 mAh/g^33^). These materials represent just a handful of those capable of delivering high specific capacities in KIBs. It is very likely that various other layered materials exist with the potential to deliver capacities comparable to those already studied. A comprehensive literature search identifies As_2_S_3_, found naturally as the mineral orpiment,^34^ as a previously unexfoliated layered material. It is a compositional analogue of the well-known and previously exfoliated 2D materials^35^ Sb_2_S_3_ and Bi_2_S_3_ and has significant potential for ion storage in battery electrodes (theoretical capacity = 1305 mAh/g, see below). However, to our knowledge, no reports exist on using it in battery electrode applications.

Although arsenic is indeed toxic, especially in its inorganic, trivalent form,^36^ it is still widely used in a range of industries including textiles, wood preservation, semiconductor growth, and the production of lead-acid batteries.^37^ In any case, irrespective of the possibility for its use in real applications, from the perspective of basic science, it is important to study its potential as an ion-storing electrode material. Indeed, such studies have previously been carried out on both elemental arsenic^38^ and arsenic-containing compounds.^39^

In this study, we explore As_2_S_3_ nanosheets as an anode material for KIBs. We demonstrate that orpiment can be easily exfoliated in liquids to give nanosheets in reasonable quantities. Using single-walled carbon nanotubes as binders, As_2_S_3_ nanosheets can easily be solution cast into films for use as K-ion anodes. Electrochemical characterization shows these anodes to demonstrate capacities as high as 619 mAh/g and exceptional capacity retention over 1000 charge–discharge cycles.

## Results and Discussion

### Few-Layered As_2_S_3_ Exfoliation and Characterization

**Figure 1:**
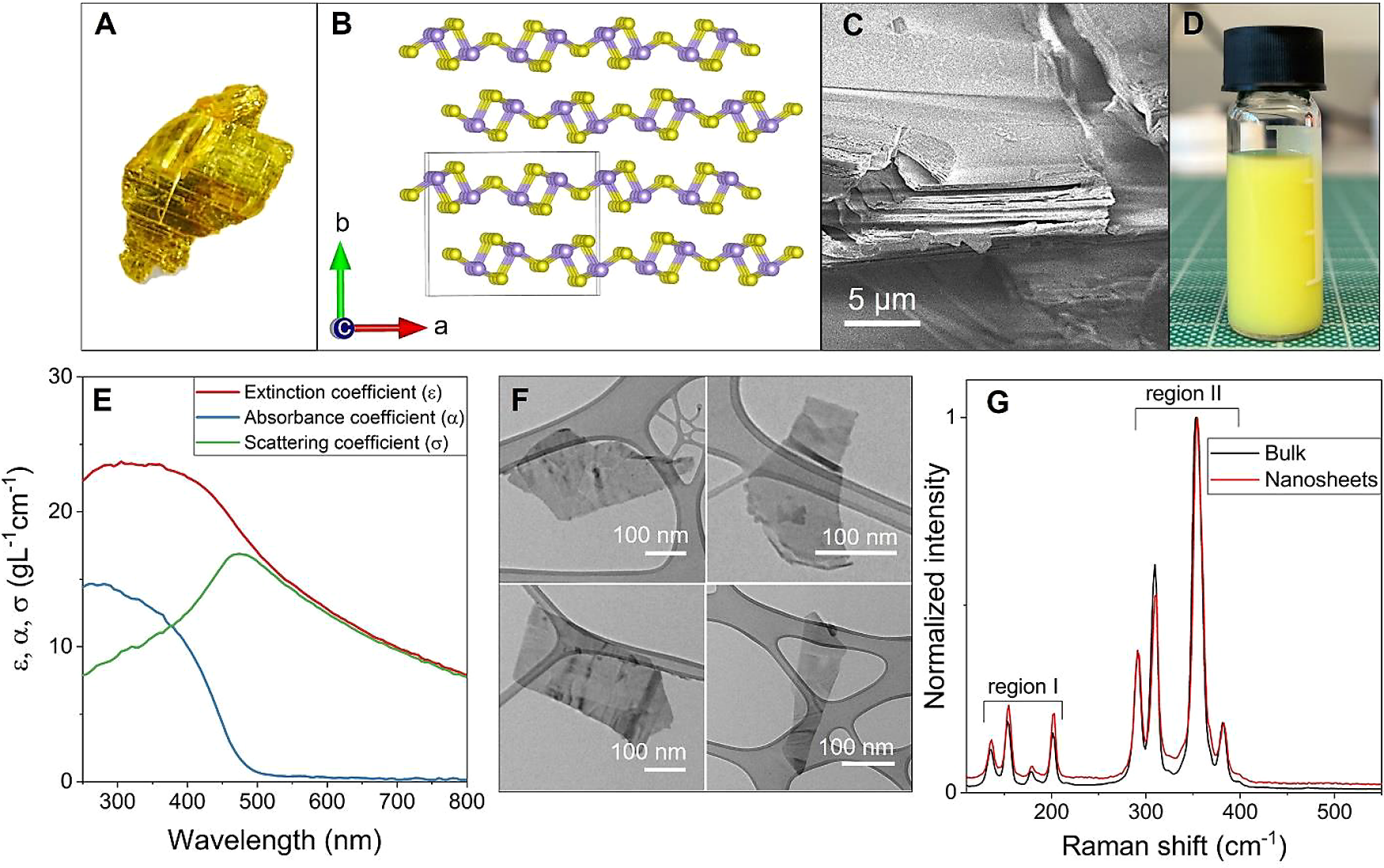
Characterization of liquid-phase exfoliated As_2_S_3_. (A) Photograph of the mineral orpiment. (B) Crystal structure of bulk As_2_S_3_, composed of covalently bonded ruffled sheets of As_2_S_3_ oriented in the (010) direction, held together by weak van der Waals forces. Arsenic and sulfur atoms are depicted as purple and yellow spheres, respectively. The unit cell is represented with a black box and consists of 2 layers with a total of 20 atoms. (C) SEM image of the orpiment crystal surface (as shown in A) displaying multilayered sheets. (D) Dispersed As_2_S_3_ nanosheets in solvent 2-propanol obtained after liquid-phase exfoliation in an inert atmosphere. (E) Optical extinction, absorbance, and scattering coefficient spectra of the dispersion versus wavelength. (F) TEM images of As_2_S_3_ nanosheets. (G) Raman spectra comparison between bulk and exfoliated As_2_S_3_.

As_2_S_3_ exits as a naturally occurring layered crystal, orpiment, as shown in Figure 1A.^40,41^ These yellowish crystals belong to the family of 2D layered materials.^40,41^ The crystal structure of As_2_S_3_ belongs to the space group P2_1_/n and has a monoclinic lattice structure.^40−44^ Each single layer is made up of AsS_3_ pyramids linked together by strong As–S–As covalent bonds with weak van der Waals interlayer interactions as shown in Figure 1B.^45^ The unit cell contains 20 atoms distributed over two monolayers (Figure 1B).^41,44,45^ An SEM image of the orpiment crystal surface, as shown in Figure 1C, confirms the layered morphology.

An orpiment crystal was first cleaned and converted to powder via bath sonication in the solvent 2-propanol (details of cleaning and liquid-phase exfoliation process are given in the Methods section). Inert atmosphere liquid-phase exfoliation (LPE) was performed by bath sonicating the resultant powder for 10 h in dried, distilled, and degassed 2-propanol under an argon atmosphere. The resultant dispersion was centrifuged to remove a large unexfoliated material (220 g) and again to remove any very small particles (3800 g) resulting in a standard dispersion (Figure 1D). The concentration (*C*) of the dispersion was determined to be 1 mg/mL via filtration of 1 mL of dispersion and mass weighing.

To investigate the optical properties of the dispersed nanosheets, optical extinction spectroscopy was employed, as shown in Figure 1E. The extinction spectrum (Ext = −log *T*) was obtained and subsequently converted into an extinction coefficient spectrum (ε = Ext/*Cl*, where is *l* is the cuvette length).^46−49^ As depicted in Figure 1E, the extinction coefficient spectrum exhibits a steady increase with decreasing wavelength with no clear optical absorption band-edge. The lack of a clear band-edge in nanosheet dispersions is generally due to light scattering.^48^ To address this, scattering coefficient spectra (σ) were measured (Figure 1E) using an integrating sphere and then subtracted from the extinction coefficient spectrum (ε) to obtain the absorption coefficient spectrum (α).^46,48,49^ The α-spectrum shows a clear band-edge at λ = 500 nm, corresponding to an optical gap of 2.5 eV. This is consistent with bandgap values of ∼2.5 eV associated with As_2_S_3_, as reported in the literature.^41,42,44^

Furthermore, transmission electron microscopy (TEM) was utilized to characterize the dispersion. TEM images, as presented in Figure 1F, reveal the presence of As_2_S_3_ nanosheets with a mean length (<*L*>) of approximately 350 nm. Raman spectroscopy was also employed to characterize the intermolecular vibrations. Figure 1G presents the Raman spectra of both bulk and exfoliated As_2_S_3_. These spectra exhibit distinct, well-defined sharp phonon modes, which can be categorized into two distinct regions: region I spans from wavenumber 120 to 210 cm^–1^, while region II encompasses wavenumber from 280 to 390 cm^–1^. Raman phonon peaks in region I at 135, 154, 179, and 202 cm^–1^ correspond to the bond-bending vibrations, while the phonon peaks in region II at 291, 310, 354, and 382 cm^–1^ correspond to bond-stretching vibrations.^43−45^ The phonon modes of bulk and exfoliated As_2_S_3_ are the same within the resolution of the Raman spectrometer used in this study and are in alignment with literature data,^41,43,44^ showing that exfoliation does not change the material structure. In addition, no extra Raman modes were detected, confirming that As_2_S_3_ nanosheets are free of impurities and did not undergo oxidation during the exfoliation process.

**Figure 2:**
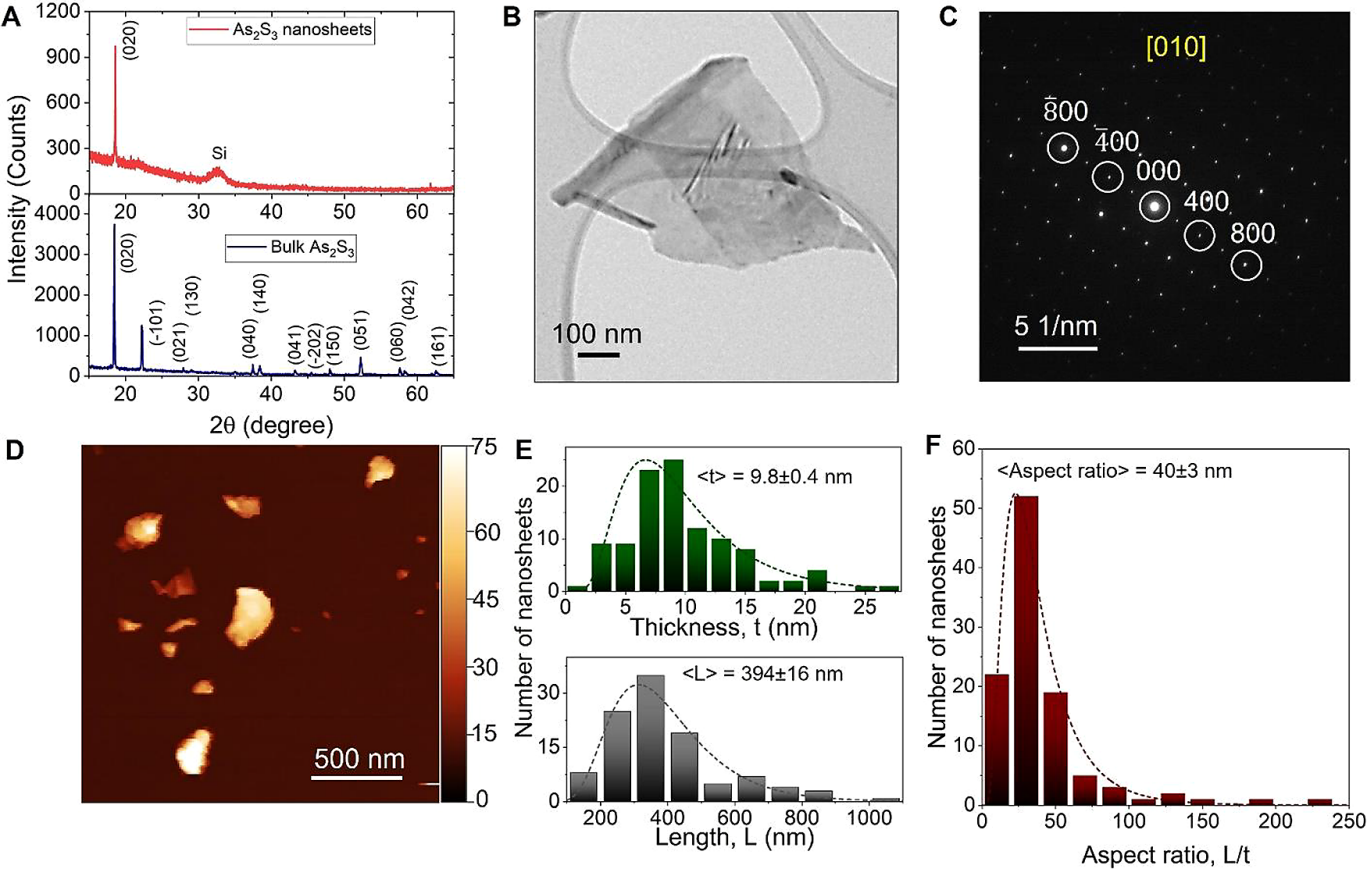
Structure and aspect ratio analysis of As_2_S_3_ nanosheets. (A) X-ray diffractogram of both the bulk crystal and exfoliated nanosheets. Diffraction peaks are indexed using PDF card number 01-071-2435 (crystal system: monoclinic, space group: P2_1_/n). (B) TEM image of an As_2_S_3_ nanosheet with the corresponding SAED pattern (C). The nanosheet is oriented along the [010] direction parallel to the As_2_S_3_ crystal cleavage plane. A full assignment of the diffraction spot pattern can be found in the Supporting Information. (D) Atomic force microscopy image of As_2_S_3_ nanosheets deposited on a substrate. (E) Distribution plots illustrating the thickness and length of nanosheets. More than 100 nanosheets were individually measured for length, width, and step height, exhibiting a log-normal distribution, as indicated by the dotted curve. The aspect ratio of each nanosheet, defined as the ratio of its length to thickness, was calculated and is depicted in the distribution plot (F). The mean thickness, length, and aspect ratio are reported in the plot, along with standard errors.

To gain an understanding of the crystal structure of As_2_S_3_ few-layered nanosheets, we employed both X-ray diffraction (XRD) and selected area electron diffraction (SAED) through TEM. Figure 2A shows the XRD pattern of the bulk crystal and exfoliated As_2_S_3_ nanosheets in the 2θ range from 15° to 65°. The diffraction pattern of the bulk crystal displayed several peaks at different angles, including 2θ = 18.5°, 22.2°, 28.0°, 29.0°, 37.5°, 38.4°, 43.3°, 45.3°, 48.1°, 52.3°, 57.7°, 57.9°, and 62.6°. These peaks were assigned to various *hkl* planes in the reciprocal lattice space of the monoclinic crystal structure of As_2_S_3_, such as (020), (−101), (021), (130), (040), (140), (041), (−202), (150), (051), (060), (042), and (161). It was observed that these peaks were closely aligned with the reference data for orpiment (PDF 01-071-2435), and no impurity phases were detected. A significant preferred orientation was observed, as the (0 2 0) diffraction peak was approximately three times more intense than the next highest peak, indicating that this plane is parallel to the alignment of layers along the *b*-axis in the orpiment crystal structure (which is also parallel to the cleavage plane). The highest intensity of this peak strongly suggests that the powdered sample particles predominantly took on a platelet shape, rather than a spherical form.^50^ The finding is in line with similar patterns that have been previously reported in the existing literature.^51^ For the exfoliated nanomaterials, the presence of only the (0 2 0) peak is a clear indication of their 2D morphology.^50^ It is worth noting that the exfoliated nanosheets are not expected to perfectly restack like the bulk material, resulting in a distinct XRD pattern compared to the bulk material;^50^ therefore, As_2_S_3_ is also expected to behave the same. The broad XRD peak at 2θ position 32.5° corresponds to the silicon (002) substrate.^52^ TEM imaging of a few-layered-thick nanosheet (Figure 2B) allowed measurement of a SAED pattern (Figure 2C), showing the presence of well-defined sharp diffraction spots. The diffraction spot pattern is fully indexed (Figure S1, Supporting Information) with the monoclinic lattice structure of As_2_S_3_ with nanosheet orientation in the [010] direction, thus representing high crystallinity of exfoliated nanosheets of As_2_S_3_. Energy dispersive X-ray spectroscopy (EDX) conducted on As_2_S_3_ nanosheets confirms the presence of As and S elements (Figure S2, Supporting Information). Notably, oxygen is absent in the elemental EDX scans, confirming that the nanosheets formed by inert-gas LPE are anhydrous. The average atomic ratio calculated over 24 randomly selected nanoplatelets reveals <S/As> = 1.5.

Additionally, we employ atomic force microscopy (AFM) to examine the distribution of length (*L*), thickness (*t*), and aspect ratio (*L*/*t*) of exfoliated nanosheets. Figure 2D shows an AFM image displaying several nanosheets with varying sizes and thicknesses. For each individual nanosheet, length and apparent thickness were measured. Apparent thickness values were converted into real thickness (*t*) by measuring the step height (Figure S3, Supporting Information) as described in detail in Section A of Supporting Information. The length and thickness distribution of 100 nanosheets is presented in Figure 2E, with the mean value represented as <*L*> = 394 ± 16 nm and <*t*> = 9.8 ± 0.4 nm, along with standard errors. Additionally, we calculate the aspect ratio of each nanosheet by taking the ratio of length to its thickness and plot as shown in Figure 2F. The mean aspect ratio is found to be 40 ± 3, which is at the upper end for nanosheets obtained from LPE of 2D materials,^48,53^ implying that the ratio of fracture (in-plane) to delamination (out-of-plane) energies is relatively large for this material.^53^

### Electrochemical Storage of K^+^ Ions in As_2_S_3_ Anodes

Previous research has consistently shown that utilizing active materials in the form of 2D nanosheets within lithium- or sodium-storing battery electrodes leads to near-theoretical capacities.^25^ This is especially the case when single-walled carbon nanotubes (SWCNTs) are used as a replacement for both conductive additives and polymeric binders.^54^ In this composite electrode architecture, SWCNTs establish a mechanically robust and electrically conductive network that enhances charge delivery.^54,55^ This, in turn, allows for the optimization of capacity, rate capability, and cyclability.^54^ We anticipate that the success of this architecture for lithium- and sodium-storing electrodes can be extended to potassium storage. Due to its lower density, we might expect As_2_S_3_ to yield a higher specific capacity for potassium-storing anodes as compared to its analogues Sb_2_S_3_ (*C*_theory_ = 946 mAh/g) and Bi_2_S_3_ (*C*_theory_ = 625 mAh/g).^32,33^ However, to our knowledge, there are no reports of Li-, Na-, or K-storing electrodes fabricated from As_2_S_3_. Here, we will explore the potential of nanocomposites consisting of LPE-produced As_2_S_3_ nanosheets and SWCNTs for developing high-performance K-ion anodes.

**Figure 3:**
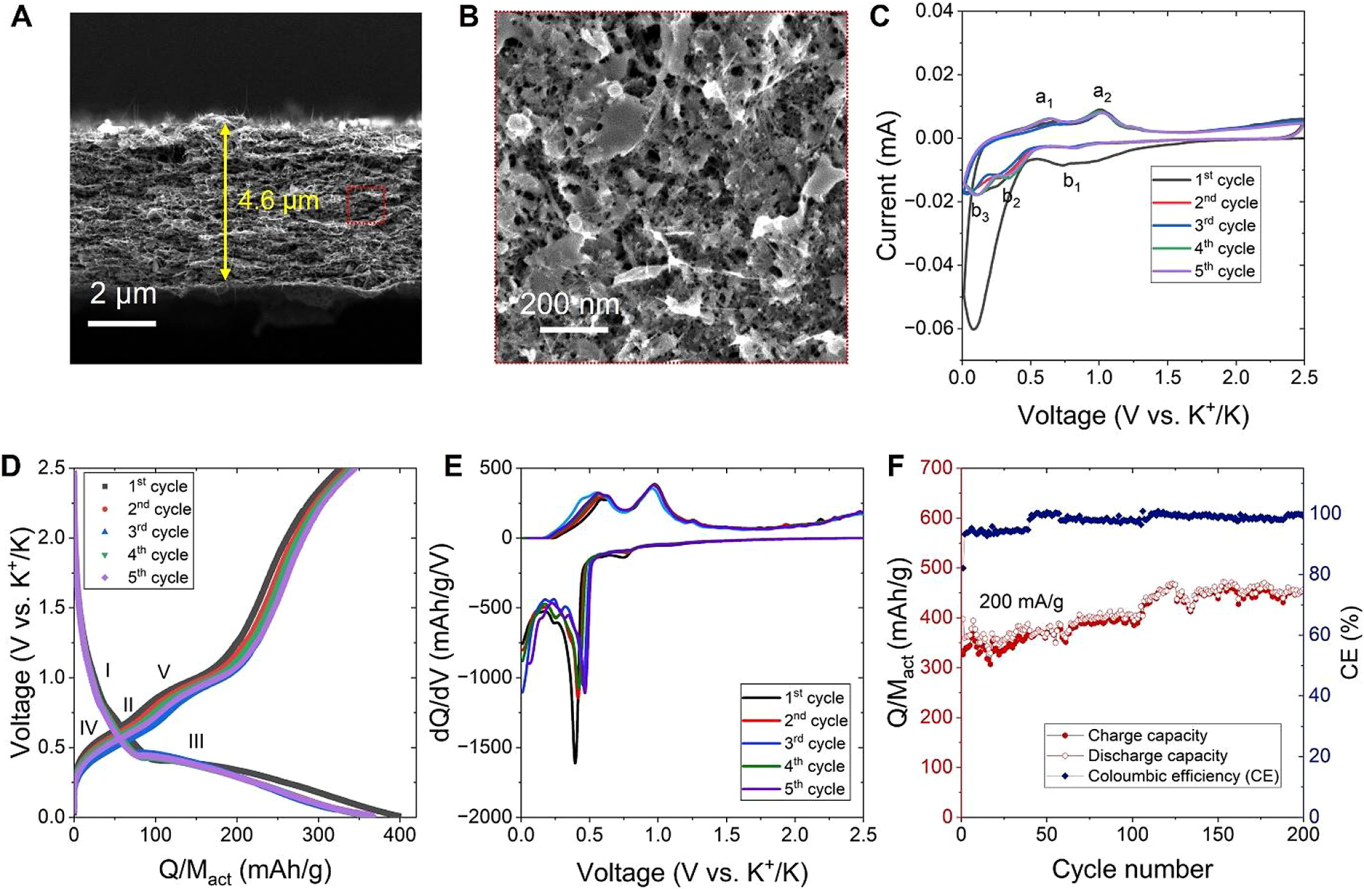
Investigation of the electrochemical mechanism in few-layered nanosheets of As_2_S_3_ (70 wt %) and CNT (30 wt %) nanocomposite anodes for KIBs. (A) Cross-sectional SEM image of the As_2_S_3_ electrode with a mass loading of 0.44 mg/cm^2^ and a thickness of 4.6 μm. (B) SEM image revealing the interconnected network of nanosheets and carbon nanotubes on the electrode surface. (C) Initial five cycling voltammetry (CV) curves acquired at a scan rate of 0.1 mV/s. (D) Voltage profiles for the galvanostatic charge–discharge (GCD) for the first five cycles recorded at a current density of 200 mA/g and the corresponding differential voltage plots (E). (F) Charge–discharge cycling and Coulombic efficiency performance of the electrode at 200 mA/g, demonstrating an increase in specific capacity (normalized to the active mass represented as *Q*/*M*_act_) with cycle number, plateauing after 124 cycles.

Nanocomposite anodes comprising As_2_S_3_ nanosheets and SWCNTs were fabricated for use in a half-cell configuration. In short, the dispersion of As_2_S_3_ nanosheets was mixed with an SWCNT dispersion in 2-propanol and then subjected to vacuum filtration onto a Celgard 2320 membrane (for detailed procedures, please refer to the Methods section). This process led to the creation of free-standing nanocomposite films (as shown in Figure S4 of Supporting Information) containing 28–30 wt % nanotubes, without the need for a polymeric binder. Subsequently, the free-standing films were cut into the required dimensions with an area of 0.178 cm^2^ for electrochemical testing. The SEM image in Figure 3A, displaying the cross-sectional view of the free-standing As_2_S_3_@CNT electrode (total mass loading of 0.44 mg/cm^2^), reveals a thickness of 4.6 μm. A magnified view of the electrode’s surface, as depicted in Figure 3B, reveals a well-connected porous network of 2D nanosheets interlinked with 1D carbon nanotubes. The SEM cross-section image of the composite film, along with the corresponding elemental composition maps, is presented in Figure S4. The EDX spectra of the composite electrode indicate the presence of As and S elements. Elemental maps demonstrate a uniform distribution of As and S elements, with an expected stoichiometry of As_2_S_3.1_. The electrode porosity was calculated by estimating its density using the thickness and mass loading data, resulting in a density of 0.95 g/cm^3^. Given the densities of As_2_S_3_ and SWCNT, which are 3.43 and 1.8 g/cm^3^, respectively, the calculated porosity of the electrode is approximately 67%, similar to but slightly higher than nanosheet-only networks.^56^

Electrochemical characterization was carried out to assess the utility of these electrodes for potassiation/depotassiation. Figure 3C depicts the cyclic voltammograms (CV) for the first five cycles acquired at a scan rate of 0.1 mV/s in the voltage window between 0.01 and 2.5 V. The CV curve indicates that during the first discharge process, a broad cathodic peak appears at 0.75 V, mainly due to the formation of solid–electrolyte interface (SEI) films. This peak disappears in the subsequent sweep cycles. Another peak is seen at 0.08 V, which is likely associated with the alloy reactions between K and As, and is skewed by SEI formation.^57^ From the second cycle, the CV curves follow similar line shapes and completely overlap demonstrating a highly reversible electrochemical reaction. During the subsequent scans, the cathodic peaks are identified in the overlapping CV curves marked as b1, b2, and b3 and located at 0.81, 0.31, and 0.12 V, respectively. Materials with the same structure are known to belong to the same family and are expected to exhibit similar characteristics. Based on the matching stoichiometry, as well as the similar structure and shape of the CV curves, it is expected that our As_2_S_3_ electrodes will also demonstrate consistency with the materials from the same family by displaying similar ion storage mechanisms. Therefore, it is predicted that the electrochemical reaction of As_2_S_3_ with potassium ion is likely to proceed similarly to that of Bi_2_S_3_^58^ and Sb_2_S_3_.^32,59^ During potassiation, the small cathodic peak at 0.81 V (b1) is attributed to K-ion insertion into As_2_S_3_. The second reduction peak at 0.31 V (b2) is assigned to a conversion reaction, i.e., electrochemical reduction of As_2_S_3_ to arsenic with accompanying the formation of an amorphous K_2_S, and the third reduction peak centered at 0.12 V (b3) can be assigned to alloying reactions between K and arsenic. In addition, during depotassiation, two anodic peaks appearing at 0.64 V (a1) and 1.0 V (a2) can be assigned to dealloying and further reversible conversion and deintercalation reactions, respectively.^31−33,58,59^

To check the validity of the proposed mechanism, we performed galvanostatic charge–discharge (GCD) experiments on an As_2_S_3_@CNT electrode. Here, both charge and discharge capacities, as well as the current density, were calculated based on the active material (As_2_S_3_) mass and referred to as *Q*/*M*_act_. In this study, as a part of the initial activation procedure, all electrodes were first subjected to 10 cyclic voltammograms at a scan rate of 0.1 mV/s. This is a reasonably standard activation procedure that allows the formation of an SEI layer on the electrode surface. After the activation cycles, we continued cycling the electrode for 200 charge–discharge cycles at a higher current of 200 mA/g. The voltage profiles collected for the first 5 cycles are displayed in Figure 3D with a voltage range of 0.01–2.5 V. During the discharge process, three plateau regions are noticed in the voltage range of 1.2–0.8 V (referred to as region I, indicating the intercalation process), 0.75–0.45 V (referred to as region II, indicating the conversion process), and 0.45–0.07 V (referred to as region III, indicating the alloying reaction). During the charging process, plateau region IV (0.2–0.75 V) indicates reverse conversion, while plateau region V (0.8–1.2 V) indicates reverse deintercalation reactions. The plateau regions align reasonably well with the peaks observed in the CV curves (Figure 3C).

Figure 3E shows differential voltage plots for the first 5 cycles, which are nearly identical to the peaks observed in the CV curves but slightly more well-defined. In the first cycle, the GCD curve at 200 mA/g delivers a discharge capacity of 398 mAh/g and a charge capacity of 327 mAh/g. In the subsequent cycles, charge–discharge curves are stable and remain nearly identical, indicating good stability of the nanostructures as anode materials. Figure 3F illustrates the resulting charge–discharge capacities of the As_2_S_3_@CNT electrodes for 200 cycles along with the Coulombic efficiencies (CEs). We consider the contribution from carbon nanotubes to the overall measured capacity of As_2_S_3_@CNT composite electrodes to be negligible due to their very low specific capacity (∼30 mAh/g), as illustrated in the Supporting Information (Figure S5). At the beginning of cycling, the electrodes achieved a CE of 82%. During the following cycles, both the charge and discharge capacities, as well as the CE, gradually increased before reaching their maximum at around 124 cycles. At this point, the discharge and charge capacities reached 468 and 466 mAh/g, respectively, with a CE exceeding 99%. Beyond this cycle, the CE remained consistently above 99%, while the discharge and charge capacities leveled off at 459 and 457 mAh/g, respectively (refer to Figure S6, Supporting Information for the voltage profiles). These findings suggest that contrary to what was previously assumed, the electrode’s activation was not fully accomplished within the first five cycles but rather necessitated over 100 cycles to reach its maximum capacity. Similar behavior was also observed by Zhou et al.^60^ in their study of a KIB anode composed of VS_2_ nanosheets. At 500 mA/g current, the specific capacity of the nanostructured VS_2_ anode increased from 290 to 360 mAh/g after 100 cycles. However, they attributed this phenomenon to increased wetting of the electrode film by the electrolyte during cycling.^60^ The cycling profile at a low current density displayed in Figure 3F demonstrates fluctuating data. This is due to the dissolution of polysulfides (that are formed during the deep potassiation process in the majority of metal sulfides) in the electrolyte and its shuttle effect results in the loss of active materials, leading to poor reversibility and fluctuating CE. Therefore, this is a significant issue associated with metal sulfides as anodes for KIBs.

**Figure 4:**
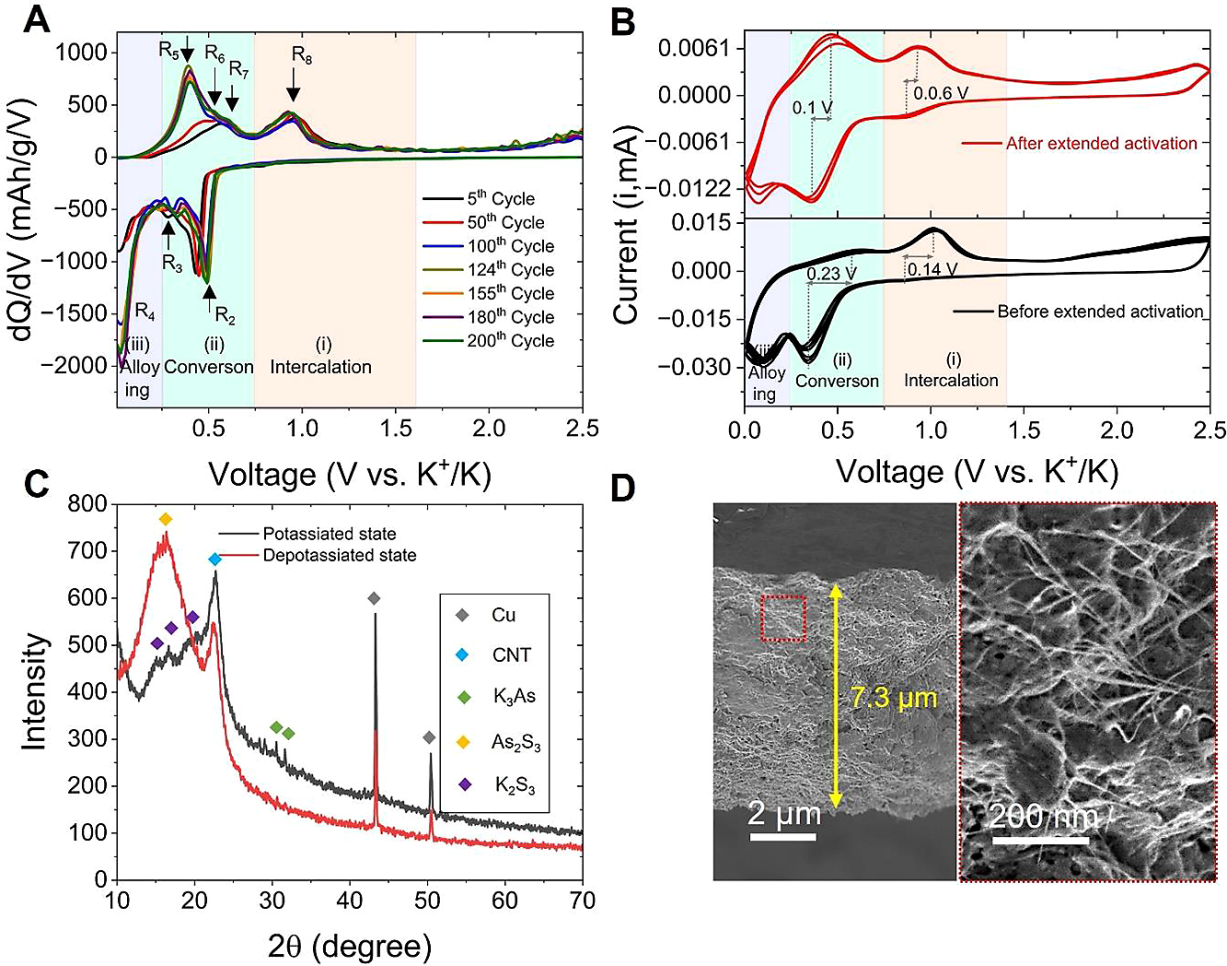
Postcycling analysis for studying the effect of extended activation on As_2_S_3_ (70 wt %) and CNT (30 wt %) nanocomposites as KIB anodes. (A) Differential voltage curves collected during the extended activation processes at a current density of 200 mA/g for 5th, 50th, 100th, 124th, 155th, 180th, and 200th cycles. (B) CV curves of the As_2_S_3_/CNT composite electrode before and after the extended activation processes. (C) XRD pattern of As_2_S_3_/CNT composite anodes in the potassiated and depotassiated states. (D) SEM images of the As_2_S_3_/CNT composite electrode after the extended activation processes.

To gain a thorough understanding of how activation leads to an increased capacity during cycling, the differential voltage plots were analyzed. Such plots are obtained by differentiating the GCD curves (i.e., those used to prepare Figure 3F) and plotted as dQ/dV versus voltage. Figure 4A shows the dQ/dV versus voltage plot for selected cycles for the 5th, 50th, 100th, 124th, 155th, 180th, and 200th at a current density of 200 mA/g. The curves collected at different cycles show a noticeable difference compared to the curve collected at cycle 5. However, after the 124th cycle, all curves overlap each other. This indicates that the activation process is complete after the 124th cycle, and the material remains electrochemically stable thereafter.

Following recent research by Nithya et al.,^58^ we propose that the electrode undergoes the following reactions at various stages of potential, which we divide into three regions as shown in Figure 4A:

During potassiation:

*Region I*: at 1.4–0.75 V (intercalation process)
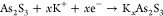
1

*Region II*: at 0.75–0.25 V (conversion reactions)
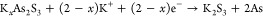
2
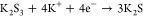
3

*Region III*: at 0.25–0.01 V (alloying reactions)
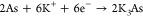
4

Overall, this reaction is equivalent to
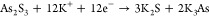
5

In Figure 4A, two redox peaks are observed in region (ii), indicating that the conversion reaction occurs in two main steps.^61^ The first step (eq 2) involves the creation of amorphous arsenic nanoparticles by reducing As_2_S_3_ into As and forming K_2_S_3_ polysulfide (peak R_2_ in Figure 4A). These nanosized As particles possess a robust ability to effectively trap the discharge products, thereby preventing the shuttling of byproducts in the electrode surface.^62^ Consequently, the subsequent reactions (eqs 3 and 4) are accelerated, which is expected to enhance the formation of K_3_As; this is clearly seen in Figure 4A as the R_4_ peak corresponding to K_3_As alloy intensifies with cycling, remaining stable after 124 cycles. During the charging process, which is also known as depotassiation, the reactions take place reversibly, and the discharge products are consumed to regenerate the original materials. Figure 4A clearly displays these reactions; peak R_5_ indicates dealloying at 0.39 V, while the R_6_ and R_7_ peaks show reverse conversion reactions at 0.48 and 0.58 V. Peak R_8_ represents the deintercalation reaction at 0.96 V. In addition, we notice some peak shifts in the dQ/dV curves, and the sharpening of the dealloying peak during the activation has a substantial influence on enhanced charge storage.

To validate our findings, postmortem analysis of As_2_S_3_ battery electrodes was performed, and the results have been presented in both Figures 4 and S6. To begin, we examined the CV curves of the As_2_S_3_@CNT anodes before and after 200 activation cycles to quickly detect any changes in their electrochemical storage properties. The analysis revealed significant changes, as shown in Figure 4B. After extended activation, the voltage hysteresis between conversion and deconversion peaks is reduced to 0.1 V, while intercalation and deintercalation peaks are reduced to 0.06 V. This leads to better reversible electrochemical charge storage, resulting in an enhanced electrochemical K-ion storage capacity. The findings suggest that the discharge products (K_2_S_3_ and K_2_S) can be efficiently trapped by highly active, ultrafine As particles formed in eq 2, which significantly reduces the shuttle effect and leads to excellent K-ion charge storage.^63^ Thus, we predict that during activation, amorphous arsenic nanoparticles play a vital role in realizing enhanced charge storage in the charge/discharge process. To evaluate the structural changes in the activated electrode, we performed ex situ X-ray diffractograms in the potassiated and depotassiated states of the electrode (Figure 4C). In the potassiated state, the electrode displayed XRD peaks corresponding to the presence of K_3_As and K_2_S_3_ phases, whereas in the depotassiated state, the XRD pattern showed peaks corresponding to only As_2_S_3_ and CNT, indicating that the structure of the As_2_S_3_ in As_2_S_3_@CNT anodes remains unchanged after cycling. This confirms the above redox reactions of As_2_S_3_ with K ions,^61^ implying that a charge equivalent to 12 electrons can be stored per formula unit of As_2_S_3_. Thus, the theoretical capacity of As_2_S_3_ is calculated to be 1305 mAh/g.

To investigate the morphological changes in the activated electrode, we employed SEM imaging. As depicted in Figure 4D, the cross-sectional view of the electrode revealed a clear expansion of from 4.6 to 7.3 μm after 200 cycles. This expansion indicates a reduction in density from 0.95 to 0.6 g/cm^3^, which in turn implies an increase in porosity from 67 to 79% and internal surface area. After cycling, the electrodes appear smoother with no visible 2D platelets indicating a uniform and amorphous structure. It is observed that the conversion-type materials undergo shape alteration upon cycling, and it is highly unlikely for them to retain their original morphology after cycling (Figure 4D). The ex situ XRD pattern of the electrode, which was activated for 200 cycles (see Figure S7), revealed a distinctive broad diffraction peak located at diffraction angles 2θ between 10° and 21°. The presence of this broad peak is a compelling indicator of medium-range ordering in noncrystalline structures of As_2_S_3_.^64^ This confirmed the transition of As_2_S_3_ from the crystalline to noncrystalline phase. It is worth noting that the observed phenomena are in line with the established literature that highlights the amorphization process occurring in both alloy and conversion-type materials during extended cycling, ultimately resulting in the formation of amorphous materials.^65^ After 200 cycles, SEM-EDX elemental maps were used to analyze the distribution of As and S elements in the cross-sectional film of the As_2_S_3_/CNT composite electrode to demonstrate its structural stability (Figure S8). After cycling, the electrodes exhibited the distribution of As and S elements, as well as the distribution of K, P, and F from the electrolyte.

**Figure 5:**
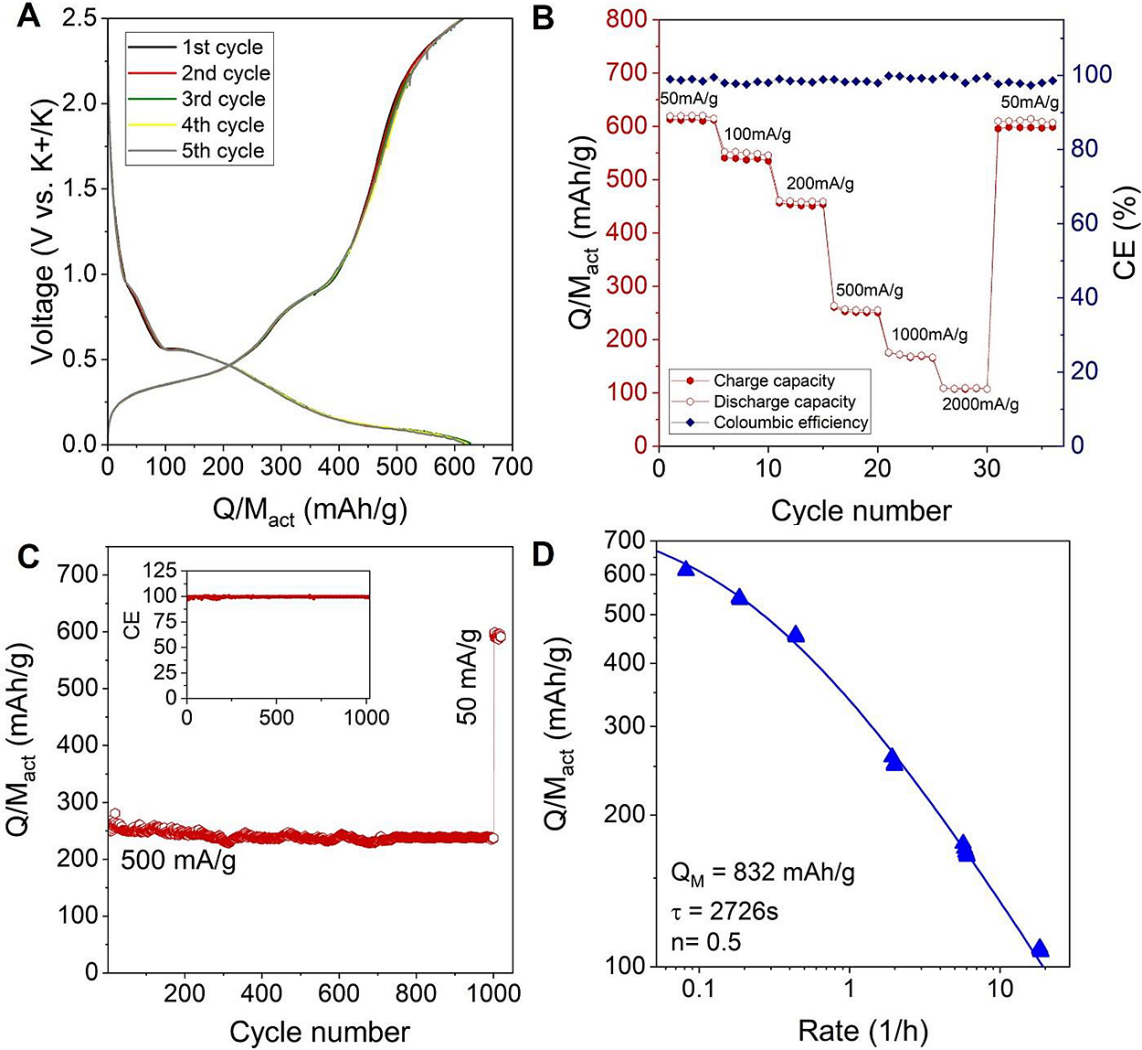
Electrochemical properties of the few-layered As_2_S_3_@CNT electrode. (A) Discharge and charge curves at 50 mA/g for the initial five cycles after extended electrode activation. (B) Rate capability of the electrode at different current densities. (C) Long cycling performance at 500 mA/g. The inset shows the CE (%) with cycle number. (D) Specific capacity plotted versus rate, *R* (*R* = (*I*/*M*_Act_)/(*Q*/*M*_Act_)), for the electrode. The fit is to eq 1 with the fit parameters given in the panel.

After the 200 cycles shown in Figure 3F, we performed five charge–discharge cycles at a current density of 50 mA/g, and the resulting voltage profiles are displayed in Figure 5A. The overlapping of voltage profiles confirmed a highly reversible electrochemical reaction, signifying the electrode's ability to repeatedly transition between charge and discharge states without substantial performance degradation. Notably, we achieved an average discharge capacity of 619 mAh/g and an average charge capacity of 612 at 50 mA/g. To comprehensively evaluate the performance, rate-performance tests were performed to assess its capacity and efficiency at different current densities, as depicted in Figure 5B. The data demonstrate the expected falloff in capacity with increasing current. However, when the current density was reduced back to 50 mA/g, an average discharge capacity of 610 mA/g was obtained, indicating excellent reversibility. The impressive nature of As_2_S_3_ nanosheets lies in their ability to offer a high specific capacity and exceptional reversibility. It is important to emphasize that this low-rate specific capacity exceeds that of almost all other reported 2D materials utilized in KIB anodes across the existing literature (see Table 1, Supporting Information). The only higher values we know of are 860 mAh/g (50 mA/g) for MoS_1.5_Se_0.5_-based electrodes.^66^ Furthermore, the cell consistently maintains a CE exceeding 98% across all tested current densities, indicative of efficient charge transfer and minimal side reactions. This underscores the cell’s capability to mitigate parasitic reactions and prevent capacity degradation.

**Figure 6:**
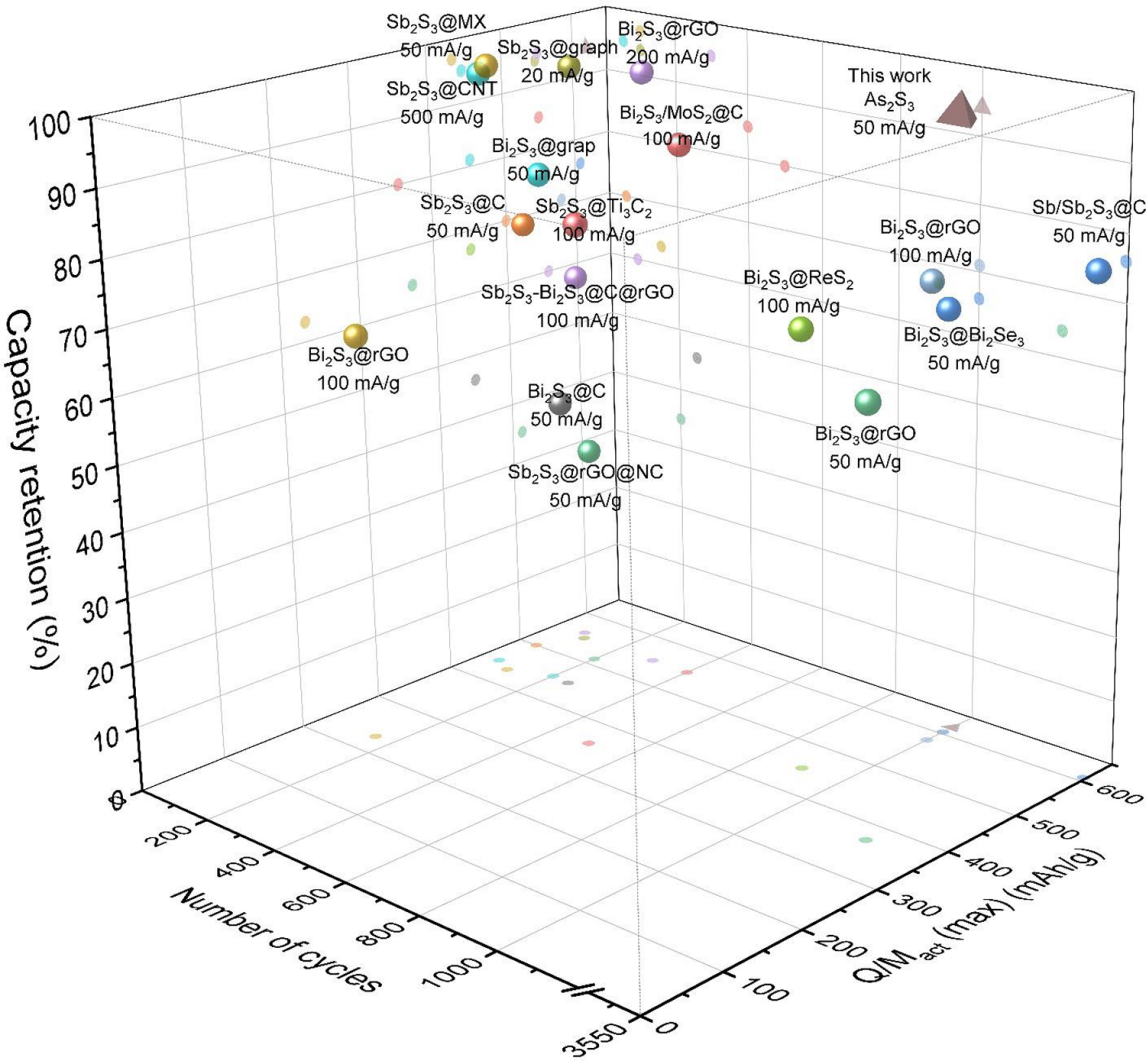
Comparative analysis of maximum specific capacity (*Q*/*M*_act_ (max)), cycle number, and capacity retention between the finding of this study and those reported for Sb_2_S_3_ and Bi_2_S_3_ in the literature. For list of references, refer to Table S2 in the Supporting Information.

To assess the cell durability, we examined capacity retention under relatively high current density conditions, subjecting the cell to 1000 charge–discharge cycles at 500 mA/g. As shown in Figure 5C, the initial discharge capacity at the beginning of cycling was 253 mAh/g, gradually decreasing to 237 mAh/g after 1000 cycles. This demonstrated an impressive capacity retention of 94% for this electrode. Notably, this level of retention is exceptional among anodes for KIBs based on 2D materials (refer to Table S1 in the Supporting Information) and is higher than that of similar materials such as Sb_2_S_3_- and Bi_2_S_3_-based KIB anodes (refer to Table S2 in the Supporting Information). A detailed comparison of the stability and capacity of As_2_S_3_@CNT anodes with other 2D material-based KIB anodes is presented in Table S1 and with other sulfide-based anodes in Table S2. The electrochemical performance of our As_2_S_3_@CNT anodes is assessed in Figure 6 by plotting the maximum specific capacity (*Q*/*M*_Act_) against the capacity retention and cycle number. Our As_2_S_3_@CNT anodes exhibit competitive capacity and stability after long cycling when compared to the best-reported results for electrodes based on As_2_S_3_ analogues, Sb_2_S_3_, and Bi_2_S_3_ for KIB. These findings underscore its robust nature and enduring electrochemical performance characteristics. Moreover, when the current density was reduced back to 50 mA/g after 1000 cycles at 500 mA/g, an average discharge capacity of 593 mA/g was obtained, again indicating excellent reversibility and superior potassium storage with greater stability. To achieve sustainable, safe, and long-lasting KIBs, the use of ether-based electrolytes is promising. By using either green ether-based electrolytes or specific molecular-designed phosphate-based electrolytes, we can significantly enhance the reliability and stability of our electrodes.^67−69^

### Quantitative Analysis of Rate Performance

Previously, we have shown that one can extract information about the ultimate capacity of electrodes, as well as quantifying the rate performance, via quantitative analysis of capacity versus rate data.^70−73^ This involves plotting the measured specific capacity (*Q*/*M*_Act_) versus charge/discharge rate as shown in Figure 5D. For such analysis, we represent charge/discharge rate via a parameter, *R*, which is defined as *R* = *I*/*Q*, i.e., the specific current divided by the specific capacity measured at that current.^70,71,73−75^ This is useful because 1/*R* is a measure of the actual charge/discharge time of the electrode at a given current. This graph shows *Q*/*M*_Act_ to decay with rate (*R*) as is usually observed.^72,73^

Such data can be analyzed using various semiempirical fitting equations,^76^ such as that proposed recently by our group:^73^
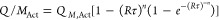
6Here, fitting yields three fit parameters: *Q*_M,Act_, which is the capacity (normalized to active mass) at an infinitely low rate; τ, the characteristic charge/discharge time; and *n*, a parameter that indicates whether diffusive (*n* = 0.5) or capacitive (electrical) (*n* = 1) limitations dominate rate performance.^70−74^ As shown in Figure 5D, this equation fits the experimental data extremely well with the following fit values: *Q*_M,Act_ = 832 ± 36 mAh g^–1^, *n* = 0.50 ± 0.01, and τ = 2726 ± 360 s. We will discuss these fit parameters one by one.

The *Q*_M,Act_ value represents the maximum achievable capacity, measured at an infinitely low rate. The fit value (832 ± 36 mAh g^–1^) was about two-thirds of the theoretical capacity (1305 mAh/g). This is somewhat lower than previous results which show that exfoliating layered materials to nanosheets which are then combined with carbon nanotubes to yield composite electrodes allows almost full utilization of the active material for storage of Li, Na, and K ions.^77−81^ However, it is consistent with our literature review data (Figure 6 and Table S2), which show Sb_2_S_3_-based K-storing electrodes to display 37–65% of theoretical capacity, although Bi_2_S_3_-based K-storing electrodes showed somewhat higher values (up to 93% of theoretical capacity).

In addition, the *n*-value is exactly equal to 0.5, indicating that our electrodes are diffusion-limited, at least for these thin electrodes (∼7 μm after cycling). Probably the most useful parameter is the time constant, τ. This represents the minimum charge–discharge time for the electrode and can be used as a metric for rate performance with higher values of τ indicating poorer rate performance. Our τ-value (2736 s) is large, comparable with the largest reported for 2D-based Li- or Na-ion batteries (ref (72) shows the reported range to be roughly 1 s < τ < 10,000 s). This indicates very poor rate performance. However, we note that rate performance in general and the time constant in particular depend very strongly on electrode thickness.^70,72,73^ Previously, we proposed that a reasonable figure of merit for rate performance is τ/*L*_E_^2^, where *L*_E_ is the electrode thickness, with large values indicating poor rate performance.^72,73^ This parameter removes some (but not all) of the thickness dependence of τ, especially for electrodes with practically relevant thicknesses (>20 μm). Taking the postcycling value of *L*_E_ = 7.3 μm yields τ/*L*_E_^2^ = 5 × 10^13^ s/m^2^. Comparing this to the summary over many Li- and Na-storing materials reported in ref (72) shows this value to be very high for this electrode thickness, indicating that As_2_S_3_ electrodes have some property that dramatically limits rate performance. Given that the value of *n* = 0.5 indicates diffusion limitations, it is suggested that the main culprit might be solid-state diffusion in the nanosheets. The time scale associated with solid-state diffusion is roughly τ_SSD_ = *L*_AM_^2^/*D*_AM_, where *L*_AM_ is the diffusion length and *D*_AM_ is the (in-plane) diffusion coefficient within the interlayer channel. Assuming that τ is dominated by the solid-state diffusion time, we can approximate τ_SSD_ = *L*_AM_^2^/*D*_AM_τ̃. Because τ is the sum of all possible time scales limiting rate performance,^73^ this equation defines the maximum possible value of τ_SSD_. Taking *L*_AM_ ∼ 200 nm (ions only need to diffuse from the edge to the center of the nanosheets), this means *D*_AM_ is slightly larger than 1.5 × 10^–17^ m^2^/s. This is somewhat smaller than the value of ∼10^–15^ m^2^/s reported for K^+^ diffusion in Sb_2_S_3_.^31^ Compared to the expected range of *D*_AM_ values for Li and Na ions in 2D materials (∼10^–19^–10^–13^ m^2^/s),^72^ these values are not particularly high, indicating that As_2_S_3_ (or indeed Sb_2_S_3_) is not best suited to achieving good rate performance. However, we note that this may not be such a problem in electrodes with practically relevant thickness (∼100 μm). Then, liquid-phase diffusion becomes dominant,^70^ rendering solid-state diffusion much less important. Such a transition was recently mapped out for sodium-storing electrodes based on red phosphorus.^79^

## Conclusions

In this study, we successfully achieved the exfoliation of layered As_2_S_3_ and fully characterized the resulting few-layered nanosheets. Combined AFM and TEM measurements showed the nanosheets to be of a high aspect ratio and of excellent quality. Raman spectroscopy and XRD analysis showed the nanosheet structure to be identical to that of bulk and confirmed the absence of impurities and oxides in the nanosheets.

Electrochemical characterization of As_2_S_3_ nanosheets as KIB anodes revealed exceptional K-storage behavior, achieving a capacity of 619 mAh/g at a current density of 50 mA/g. The electrodes demonstrated excellent cyclability, achieving a CE exceeding 99% after an initial activation process. The ex situ XRD provided support for our proposed electrochemical mechanism, affirming the storage of a charge equivalent to 12 electrons per formula unit of As_2_S_3_. This calculation indicated a theoretical capacity of 1305 mAh/g. Furthermore, our As_2_S_3_@CNT electrode displayed an outstanding capacity retention of 94% after 1000 cycles at a high current density of 500 mA/g. This combination of capacity and stability after long cycling is competitive with the very best results reported for electrodes based on As_2_S_3_ analogues and Sb_2_S_3_- and Bi_2_S_3_-based KIB anodes as displayed in Figure 6. The quantitative analysis of capacity versus rate data highlighted a close correspondence between the maximum achievable capacity (832 mAh/g) and theoretical capacity, affirming the complete utilization of active materials for ion storage. Nonetheless, we did observe a notable limitation in the rate performance, which we attributed to solid-state diffusion constraints within the nanosheets. Our analysis of electrode thickness-dependent parameters pointed to a relatively small diffusion coefficient within the interlayer channels.

We believe that the impressive theoretical capacity, high efficiency, and substantial capacity retention of these nanosheets position them as promising candidates for cutting-edge KIB applications. Future research endeavors can delve deeper into the rate performance challenges and work toward unlocking the full potential of these nanosheets for practical energy storage applications.

## Methods

### Exfoliation

The As_2_S_3_ crystal was ground into chunks of powder, which was bath-sonicated in 2-propanol for 30 min at a frequency of 37 MHz at ambient temperature. This results in a light-yellow dispersion, with chunks of powder at the bottom of the vial, which were collected for the inert LPE procedure. This step ensures the removal of any surface impurities if present. For the inert-gas exfoliation LPE process, the cleaned chunks of powder were bath-sonicated in an argon atmosphere for 10 h in dried, distilled, and degassed 2-propanol. The initial starting concentration was kept at 2 mg/mL. A round-bottom flask was used in the sonication process (Branson ultrasonic bath, CPX2800-E, 130 W). The dispersion was cooled periodically by exchanging the bath water with ice-cooled water every 30 min to maintain a temperature < 10 °C. Following sonication, the obtained dispersion underwent two consecutive centrifugation steps. The first step involved centrifugation at 220 g for 2 h aimed at removing large and thick unexfoliated particles. The supernatant obtained after this step was separated from the sediment under an inert atmosphere and subjected to a second centrifugation step at 3800 g for 3 h. This step ensures the removal of impurities and defective nanomaterials that are typically present in the supernatant at higher speeds, leaving pristine few-layered nanosheets in the sediment. The sediment was then redispersed in a fresh 2-propanol solvent, and the concentration was carefully measured. This is referred to as a standard sample.

### Electrode Formation

In the first step, a dispersion of SWCNTs was prepared in 2-propanol with the goal of achieving a concentration of <0.1 mg/mL to prevent nanotube aggregation in the obtained dispersion. To achieve this, 8 mg of P3-SWCNTs were suspended in 80 mL of 2-propanol, and the resulting solution underwent probe sonication for 3 h using a horn-tip sonic probe (Vibracell CVX, 750W). The amplitude was set at 50% with a pulse duration of 6 s on/2 s off. The temperature was carefully maintained at <10 °C throughout the probe sonication process by employing ice-cooling. The outcome of this process was a black dispersion, which was allowed to free-stand for 2 h. Afterward, the top two-thirds of the dispersion was separated, and its final concentration was measured via filtering, resulting in a concentration of 0.085 mg/mL. In the second step, the standard exfoliated dispersion of As_2_S_3_ was mixed with the SWCNT dispersion in the weight ratio of 70:30 and was bath-sonicated for 30 min to ensure uniform mixing. In the final step, the resultant mixture was vacuum-filtered using the Celgard 2320 membrane with a thickness of 20 μm and an area of 2 cm^2^. This resulted in a free-standing nanocomposite film that would be utilized as an electrode. This resultant film was subsequently cut into an area of 0.178 cm^2^, which was required for electrochemical testing. The areal mass loading of As_2_S_3_ electrodes was calculated by using the thickness obtained from SEM cross-sectional measurements, along with the area and mass of the electrode, yielding a value of 0.44 mg/cm^2^.

### Assembly of K-Ion Half Cells

K-ion half cells were assembled using CR-2032 type coin cells (14 mm; MTI Corp.) in a glovebox filled with argon, ensuring O_2_ and H_2_O levels were maintained below 0.1 ppm. Potassium metal pieces, initially soaked in mineral oil within the glovebox, were wiped clean to remove the oil. Subsequently, the metal pieces were cut and sliced with a blade to expose fresh metal surfaces and then rolled into flat discs for use as counter/reference electrodes. Whatman glass fiber filters (GD/D, diameter 90 mm) were employed as separators. The electrolyte consisted of potassium bis(fluorosulfonyl)imide (KFSI, 2 M) dissolved in triethyl phosphate (TEP). After assembly within the glovebox, the cells were removed and placed in an oven at 40 °C for 24 h before conducting electrochemical measurements.

### Characterization

The UV–visible extinction and absorption measurements were conducted using a PerkinElmer Lambda 1050 spectrometer within the wavelength range of 250–800 nm. Quartz cuvettes with a 4 mm path length were employed for this purpose. The concentration of the dispersion was determined by filtering and weighing membranes (Celgard 2320) to calculate the extinction coefficient spectra. For the absorbance spectra, a setup utilizing an integration sphere with a radius of 150 mm was employed. TEM imaging was performed using a JEOL 2100 instrument on holey carbon grids (400 mesh) with an accelerating voltage of 200 kV. High-resolution TEM and SAED were carried out using an FEI TITAN G2 80–300 microscope operated in STEM mode at 300 kV acceleration. Multiframe ADF STEM image series were recorded using the Fischione model 3000 ADF detector before rigid and nonrigid registration using the SmartAlign software from HREM Research (Tokyo).

XRD spectra were obtained with a Bruker D8 Discover high-resolution diffractometer equipped with a copper tube emitting K_α_ radiation (wavelength of 1.5406 Å) and a double-bounce Ge [220] monochromator. The spectra were acquired in the 2θ range from 10 to 80° on films prepared on silicon (100) wafers and on electrodes (fresh free-standing electrode, electrode on Cu foil after it has run 200 GCD cycles, and electrodes which are potassiated and depotassiated to different voltages).

Raman spectroscopy was conducted using a Horiba Jobin Yvon LabRAM ARAMIS instrument in ambient conditions, both on bulk crystals and films made on silicon wafers by a drop-cast method using dispersion containing nanosheets. A 532 nm laser with adjustable power was used to excite the samples with a 100× objective. A grating with 1800 grooves per millimeter dispersed the signal onto the CCD detector maintained at −70 °C. Samples were prepared by drop-casting dispersion onto silicon wafers, which are heated at 50 °C.

AFM was carried out using a Bruker multimode 8 microscope in ScanAsyst mode, employing an OLTESPA R3 cantilever. The samples for AFM are prepared by drop casting a diluted dispersion on silicon wafers and heating it at 60 °C, followed by drying using argon gas.

Scanning electron microscopy (SEM) was performed using a Carl ZEISS Ultra Plus SEM. Samples were mounted on aluminum SEM stubs using conductive carbon tabs (Ted Pella) and grounded using conductive silver paint (PELCO, Ted Pella). All images were captured at an accelerating voltage of 2 kV by using a working distance of 5 mm and a 30 μm aperture. Both the Inlens and SE2 detectors were used for imaging. EDX analysis was performed using a 20 mm^2^ Oxford INCA EDX detector. Cross-sectional SEM images of battery electrodes were taken pre- and postcycling by fracturing the free-standing electrodes. The average electrode thickness was determined by measuring the electrode thickness from SEM cross sections across the electrode.

## Supporting Information Available

SAED pattern, EDX, and step-height analysis of exfoliated nanosheets of As_2_S_3_; electrochemical performance of the SWCNT electrode, postcycling analysis of free-standing As_2_S_3_@CNT electrodes, and voltage profiles; and literature comparison of this work with others (PDF)
