# Supplementary material for: Liquid-Phase Exfoliation of Arsenic Trisulfide (As2S3) Nanosheets and Their Use as Anodes in Potassium-Ion Batteries
Source: ACS Nano. 2024 Jul 22;18(31):20213–25. doi: 10.1021/acsnano.4c03501 (PMC11308769; doi:10.1021/acsnano.4c03501)
Supplement: Supplementary file 1 — nn4c03501_si_001.pdf [file nn4c03501_si_001.pdf]

SUPPLEMENTARY INFORMATION

Liquid Phase Exfoliation of Arsenic Trisulfide (As_2S_3) Nanosheets and Their Use as Anodes in Potassium Ion Batteries

*Harneet Kaur[1], Bharathi Konkena[1,§], Mark McCrystall[1,§], Kevin Synnatschke[1], Cian Gabbett[1], Jose Munuera[1], Ross Smith[1], Yumei Jiang[1], Raman Bekarevich[2], Lewys Jones[1], Valeria Nicolosi[2], & Jonathan N Coleman[1]**

[1]School of Physics, CRANN & AMBER Research Centres, Trinity College Dublin, Dublin 2, D02 E8C0, Ireland

[2]School of Chemistry, CRANN & AMBER Research Centres, Trinity College Dublin, Dublin 2, D02W9K7, Ireland

*colemaj@tcd.ie (Jonathan N. Coleman); Tel: +353 (0) 1 8963859.

§authors contributed equally to this work

CONTENTS:

	Page no.
A: SAED, TEM-EDX and step-height analysis of exfoliated nanosheets of As_2S_3.	2-3
B: Free-standing As_2S_3@CNT electrode, post-mortem analysis of SEM-EDX, X-ray diffraction pattern and voltage profiles.	3-6
C: Literature comparison of this work with others.	6-11
References	12-15

A. SAED pattern, EDX and Step-height analysis of exfoliated nanosheets of As_2S_3.

a. Selected area-electron diffraction (SAED) pattern from a few-layer thick As_2S_3 nanosheet.

The selected area diffraction pattern (SAED) obtained from a few-layered thick nanosheet provides valuable insights into the crystallography and structural characteristics of the material. It reveals the nanosheet of As_2S_3 is oriented in the [010] direction and show the periodicity of the crystal lattice (Crystal System: Monoclinic, SPGR: P21/n).

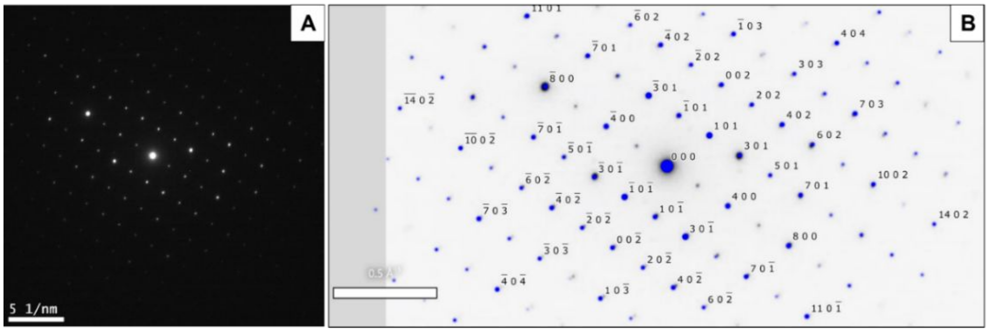

Figure S1: (A) SAED from an As_2S_3 nanosheet. (B) Assignment of the diffraction spot pattern to the monoclinic crystal system, and space group P21/n.

b. Energy dispersive X-ray spectroscopy (EDX) of As_2S_3 nanosheets for stoichiometry

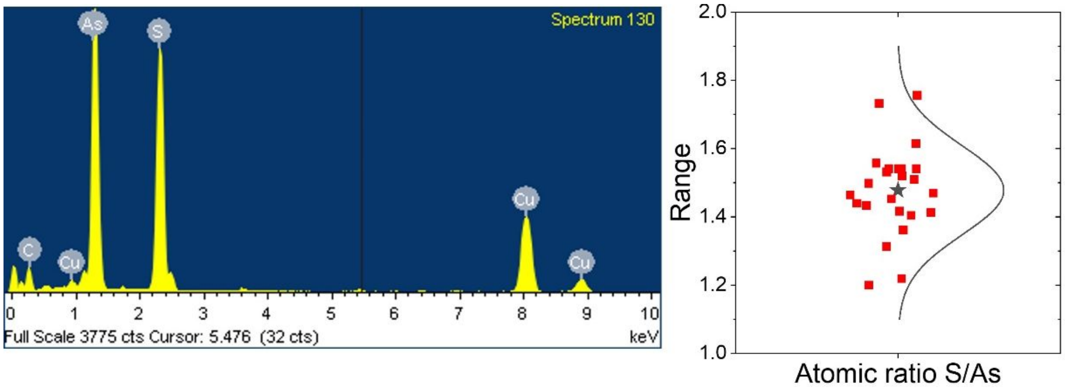

Figure S2. TEM-EDX analysis on 24 randomly selected 2D-nanoplatelets. The <S/As> ratio is in line with expectations and is plotted as a histogram.

Exfoliated As_2S_3 nanosheets were analyzed using EDX spectra, and the results are shown in Figure S2. The analysis confirmed the existence of As and S elements with an average atomic ratio of <S/As> : 1.5

c. Step-height analysis of exfoliated nanosheet.

In this context, step-height analysis pertains to the precise measurement and evaluation of the nanosheet's thickness at the atomic scale. Atomic force microscopy (AFM) is employed to accurately determine the thickness of these nanosheets. Within the AFM images, nanosheets are identified at locations exhibiting distinct steps or edges, exemplified in Figure S3. A line profile is generated across these steps using AFM software Gwyddion, and the height difference referred to as step height, is recorded. To enhance the accuracy of our analysis, measurements are conducted at multiple positions on various nanosheets, and statistical analysis was performed to find the minimum step-height. Subsequently, the actual thickness (t_{real}) of the nanosheets is derived based on these measurements, utilizing the following formula:

$$t_{real} = \frac{t_{apparent}}{Minimum\ step\ height} \times t_{monolayer}$$

The values for $t_{apparent}$ are compiled from the AFM height measurements conducted on over 100 nanosheets, while the minimum step height is determined to be 2 nm. Notably, the monolayer thickness is calculated as half of the unit cell thickness along the b-axis, which is determined to be 0.48 nm. This rigorous approach ensures accurate assessment of the nanosheet thickness.

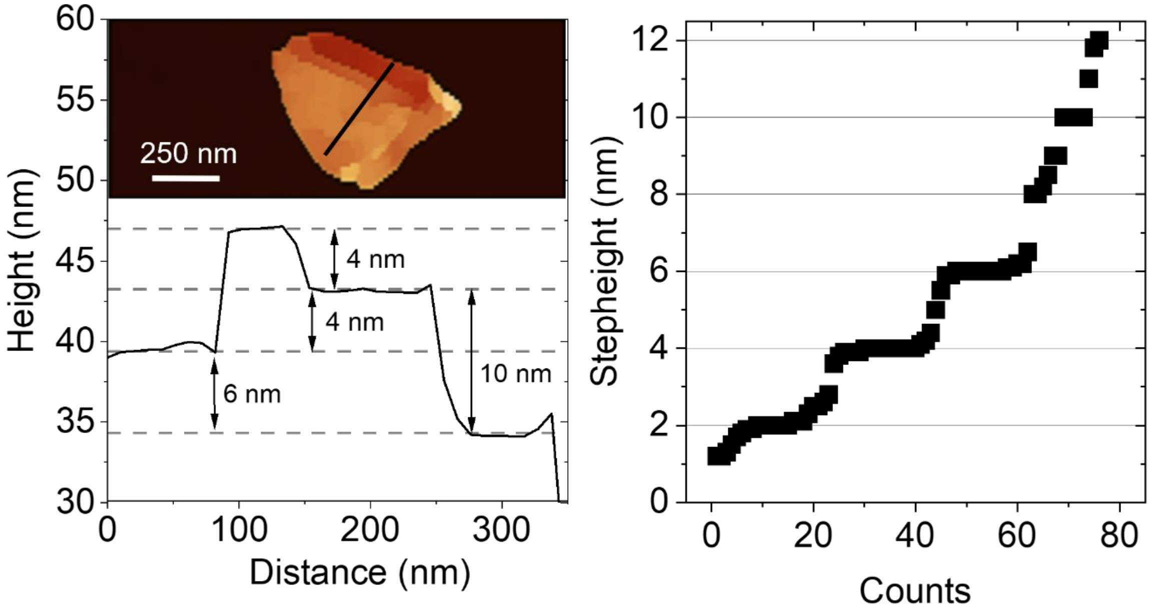

Figure S3: AFM image of an As_2S_3 nanosheet with clear steps. A line profile (black line) generated across the nanosheet display clear steps as shown, and the step height analysis over various nanosheets shows a minimum step height of 2 nm.

B: Free-standing, electrochemical performance of SWCNTs electrode, post cycling analysis of As_2S_3@CNT electrodes and Voltage profiles.

a. Free standing As_2S_3@CNT electrode

The electrode is composed of 70% by weight of As_2S_3 nanosheets and 30% by weight of SWCNT. The mass loading of the electrode is measured at 0.44 mg/cm^2. To prepare the electrode materials, the dispersions of As_2S_3 and SWCNT in solvent 2-proponal were mixed at the required weight ratio (7:3) to create the final dispersion containing 1D SWCNT and 2D-As_2S_3 nanosheets. This mixture is then bath sonicated for 30 minutes at room temperature to ensure uniform mixing, followed by filtering the final dispersion onto a Celgard membrane. Once the entire solution was filtered, the film on the membrane was allowed to dry under a vacuum pump, and subsequently stored in a glove box overnight. The resulting dried film was easily peeled off from the membrane, yielding a free standing As_2S_3@CNT electrode, as depicted in Figure S4.

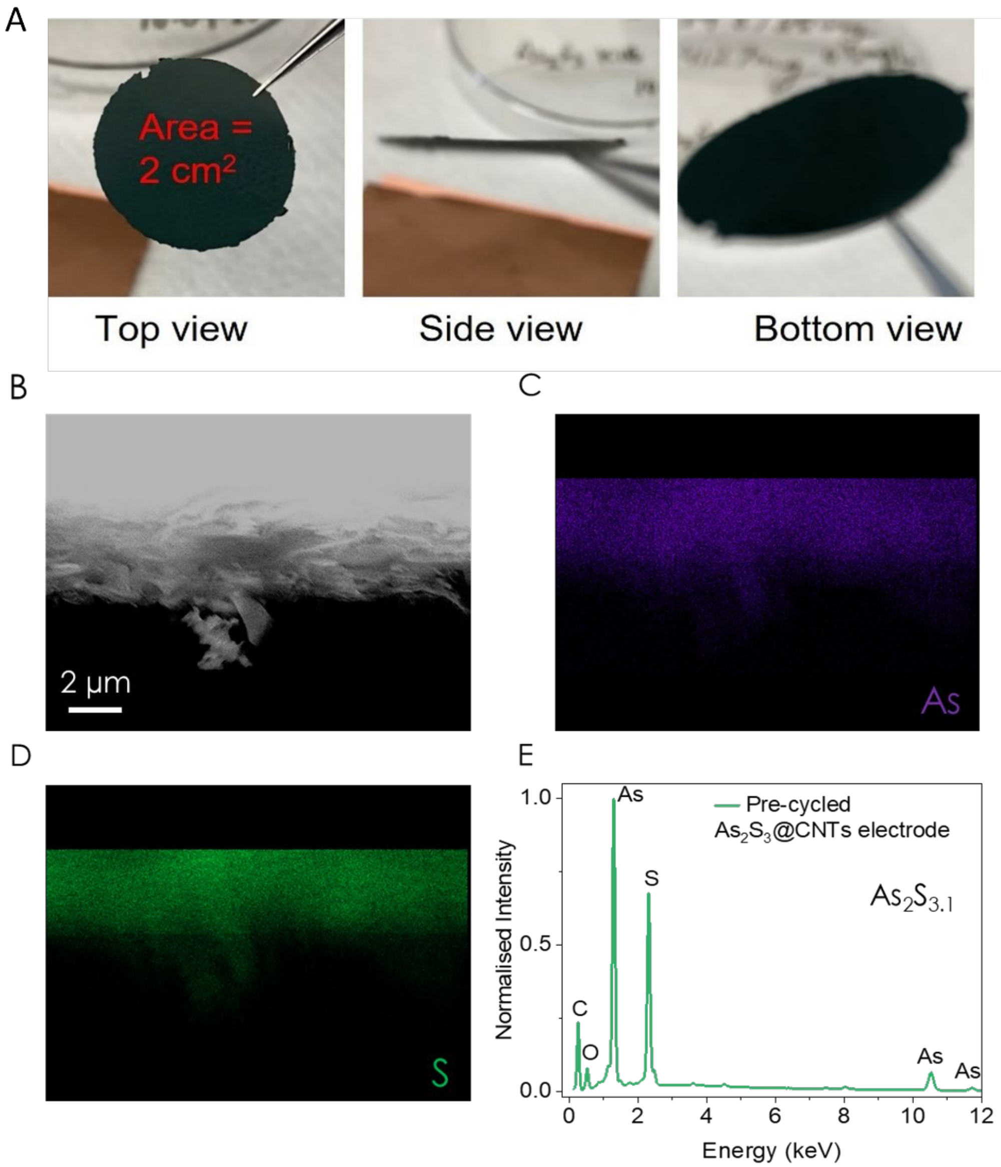

Figure S4: (A) The photograph of a free-standing As_2S_3@CNT electrode. The electrode is held with forceps and rotated to capture the top, side, and bottom views of the electrode. (B) SEM cross-section image of the composite film with the corresponding elemental composition maps, for (C) As; (D) S. (E) EDX spectra of the composite electrode, indicate the presence of As and S elements in the composite electrode. Elemental maps reveal a uniform distribution of As and S elements with an expected stoichiometry of $As_2S_{3.1}$.

b. Specific capacity of SWCNTs alone K-ion battery anode

To determine the capacity contribution of CNTs, we conducted a cycling test on SWCNTs electrodes alone at 50 mA/g for 10 cycles and 200 mA/g for 40 cycles, as shown in Figure S5. The results indicate that the specific capacity of SWCNTs for K-ion storage is approximately 32 mAh/g at 200 mA/g. As we used 30 wt% SWNTs, the maximum contribution of SWCNTs in our electrodes is 9.6 mAh/g, which is relatively small compared to the overall electrode capacity of over 450 mAh/g.

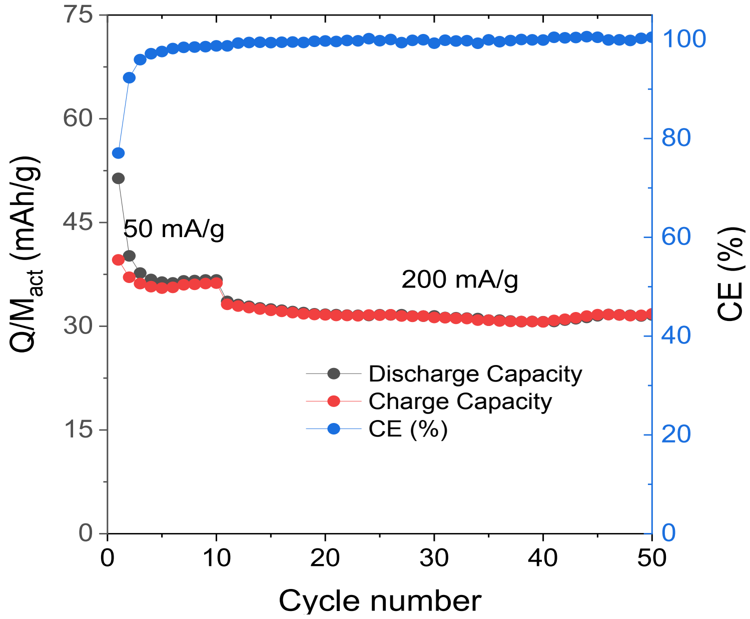

Figure S5: Cycling performance of SWCNTs film alone for K-ion storage at a current density of 50 and 200 mA/g.

c. Voltage profiles

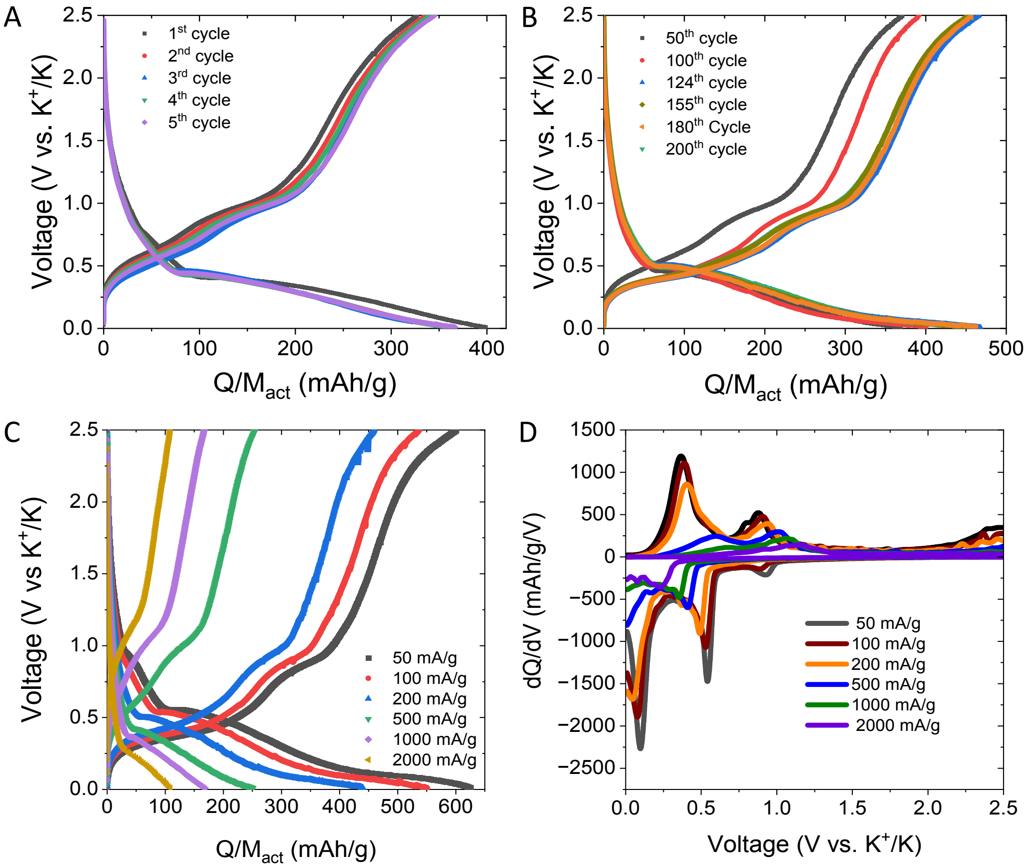

Figure S6: (A) Voltage profiles for the initial five galvanostatic charge-discharge (GCD) cycles. (B) Voltage profiles during the extended activation cycles at 50th, 100th, 124th, 155th, 180th and 200th cycles, at a current density of 200 mA/g. (C) Voltage profiles collected at different current densities and its differential voltage profiles (D). At low current density, the sharp peaks at 0.09 V and 0.36 V, indicating that highly reversible alloy and de-alloying reactions. With increasing current density, the voltage hysteresis between alloy and de-alloy reaction peaks is increasing, leading to poor charge storage at high current densities.

d. Post-mortem SEM and Ex-situ X-ray diffraction analysis of As_2S_3@CNT electrode after 200 GCD cycles.

The cell was first subjected to a ten-step process of cyclic voltammetry to establish the solid electrolyte interphase formation, followed by galvanostatic cycling at a current density of 200

mA/g for a total of 200 cycles. After this, the cell was de-potassiated to 2 V, opened and the electrode was carefully extracted for SEM and XRD measurements. The SEM image in Figure S7 demonstrates that the electrode surface appears smoother, with no apparent 2D-platelets, indicating a consistent and non-crystalline structure. It is evident that conversion-type materials experience a change in shape during cycling, making it highly improbable for them to maintain their original morphology after cycling. After 200 cycles, the SEM-EDX elemental maps were used to analyze the distribution of As and S elements in the cross-sectional film of the As_2S_3/CNT composite electrode to demonstrate its structural stability (Figure S8). After cycling, the electrodes exhibited the distribution of As and S elements, as well as the distribution of K, P, and F from the electrolyte.

The XRD pattern of the activated electrode (Figure S7) exhibited a broad peak within the 2-theta range of 10-21°, which is characteristic of amorphous As_2S_3.[1, 2] Additionally, a more distinct peak at approximately 22° corresponded to the (002) reflection of SWCNT. The presence of XRD peaks from the copper foil is due to the electrode adhering to the foil.

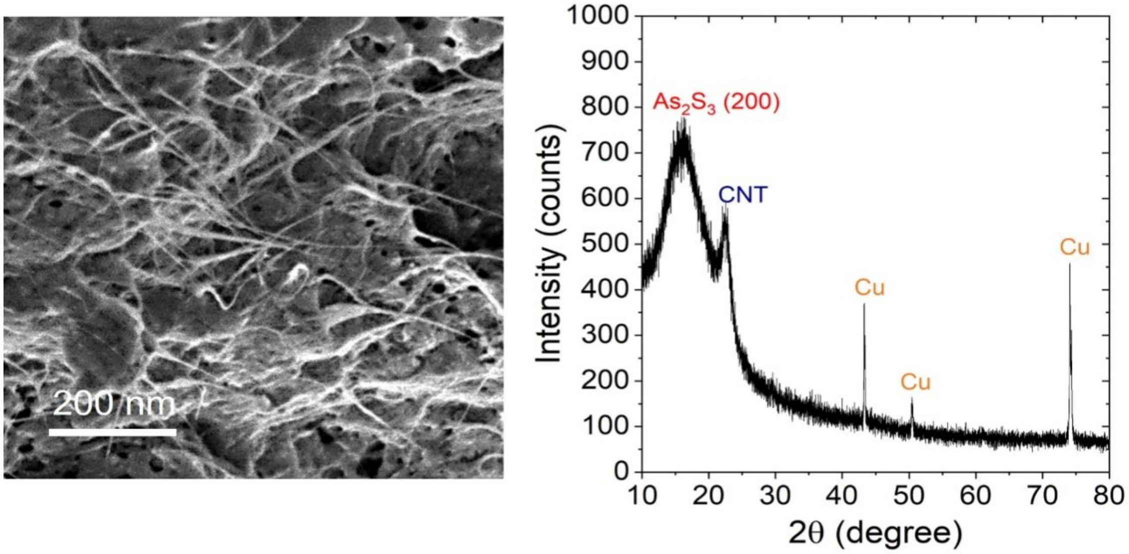

Figure S7: SEM image and X-ray diffraction pattern of the electrode after 200 charge/discharge cycles.

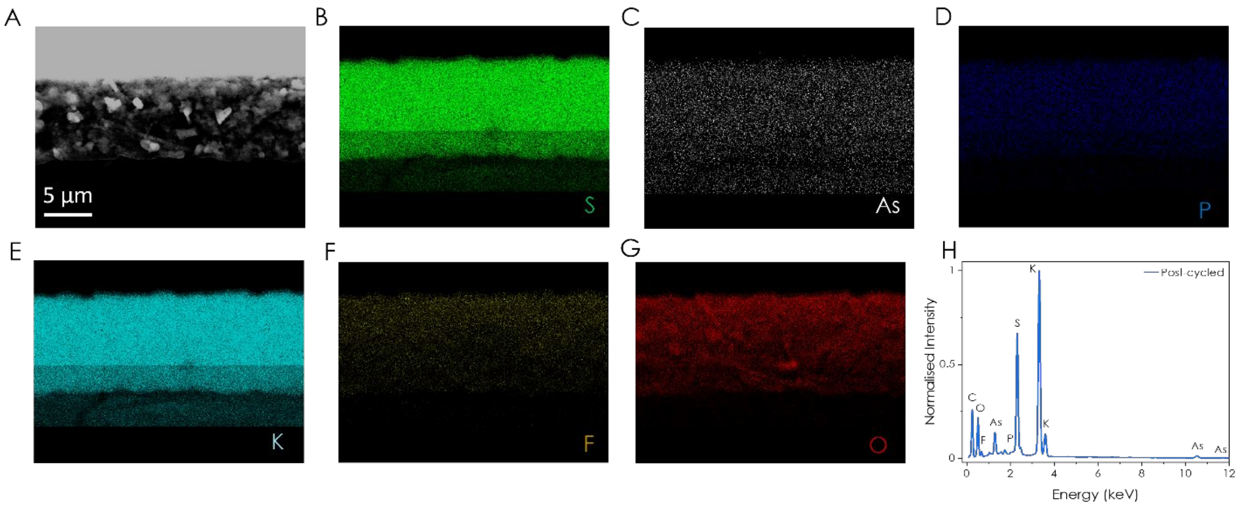

Figure S8. (A) SEM cross-section image of As_2S_3/CNT composite electrode after 200 charge-discharge cycles. The elemental mapping on the post-cycled electrode surface, for (B) S; (C) As; (D) P (E) K and (F) fluorine from the electrolyte. (G) O on the electrode surface (from SEI and oxidation after washing the electrodes). (G) EDX spectra on the post-cycled electrode represent the presence of As and S elements. Elemental maps of the electrode revealed a uniform distribution of As, S, K, P, and F elements.

C. Literature comparison of this work with others.

a. Literature comparison of As_2S_3 (this work) with other 2D materials based KIB anodes

Table S1. The literature comparison on the specific capacities, and capacity retention other 2D materials based KIB anodes of this work with other state-of-the-art latest 2D materials based KIB anodes (except for carbon/graphite).

**Anode**	**Highest stable specific capacity (mAh/g) @Current density (mA/g)**	**Number of cycles**	**Current density (mA/g) of cycling**	**Capacity at the end of cycling (mAh/g)**	**Capacity retention (%)**	**Reference**
As_2S_3@CNT	619@50<br>550@100	1000	500	237	94	This work
$FePSe_3$@CNT	472@50	500	500	223	74	[3]
$CoVMnFeZnPS_3$@graphite	500@50	1000	500	340	85	[4]
Nb_2C@rGO	352@100	1000	10000	199	83.3	[5]

Co-SLMoS_2@NOC	610@100	1000<br>3000	5000<br>5000	350<br>325	82<br>79	[6]
Ti_3C_2@C	288@100	1000	500	174	87	[7]
MoS_2-C@rGO	405@100	2000	2000	161	70	[8]
MnPSe_3@graphite	341@50	700	250	236	91.5	[9]
WS_2@SPAN	362@100	3000	1000	-	-	[10]
CNS@Ti_3C_2	306@100	1000	500	205	95	[11]
MXene@Bi_2S_3-Mo_7S_8	581@200	1000	380	276	67.1	[12]
Co_3C-MXene@C	323@100	500	500	129	67	[13]
SnTe@rGO@NC	243.9@50	50	1000	112	89.4	[14]
Sb@MoS_2@NC	390@50	80	50	210	52	[15]
FeS_2@C-rGO	526@100	500	2000	171	57	[16]
$Bi_{0.51}Sb_{0.49}$OCl@rGO	407@100	1000	100	360	88.4	[17]
MoS_2 on NC	498@200	5000	1000	399	95	[18]
MoSe_2 on NC	393@200	4800	1000	247	85	[18]
MoS_2@C	203@50	1500	500	114	80	[19]
$Ti_3C_2T_x$@NCRib	371@100	1000	1000	201.5	-	[20]
Sb_2S_3@C	500@50	200	500	400	-	[21]
WSe_2@C	393@50	500	1000	209	94.4	[22]
Nb_2C@C	398@20	200	100	257	76.2	[23]
$MoS_{1.5}Se_{0.5}$@NC	860@50	400	5000	276	80	[24]
MoS_2@MXene	290@50	100	50	206	75.9	[25]
MoSe_2@NCT	300@100	100	200	230	-	[26]
N-doped Graphene	350@50	100	100	210	78	[27]
SnS_2@MXene	342@50	800	500	206	-	[28]
Ta_2NiSe_5	308@50	1100	500	116	81.4	[29]
ZnS QD-rGO	340@50	500	1000	122	92	[30]
Sb@$Ti_3C_2T_x$	500@100	1000	1000	314	94	[31]
BP@V_2CT_x	593@100	3000	2000	261	-	[32]
$CuSbS_2$	550@100	-	-	-	-	[33]
Bi_2Se_3@C	526@50	1000	1000	214	-	[34]
CoSe_2@NC/MXene	430@100	200	500	180	60	[35]
1T-MoS_2@V_2CF_2	874@100	500	100	664	83.5	[36]
VS_2@C	488@100	50	100	436	89.3	[37]

b. *Rate performance and cycling comparison of As_2S_3 (this work) with reported Sb_2S_3, and Bi_2S_3 based KIB anodes.*

Table S2. A comparative analysis of the rate and cycling performance of As_2S_3 (this work) in comparison with Sb_2S_3 and Bi_2S_3-based KIB anodes in published studies.

**Morphology of Sb_2S_3 or Bi_2S_3 in the active material**	**Active material**	**Rate performance ($C_{specific}$ @current density (mA/g))**	**Number of cycles**	**Current density (mA/g)**	**$C_{specific}$ at the end of cycling**	**Capacity retention (%)**	**Reference**
Nanosheet	As_2S_3	619@50<br>550@100<br>459@200<br>257@500<br>171@1000<br>109@2000	1000	500	237	94	This work
Core shell nanofiber	Sb_2S_3-C@Nb_2O_5-C	350@100<br>300@200<br>275@500<br>200@1000<br>148@2000	2200	2000	96	64	[38]
Nanoflower	Sb_2S_3@Ti_3C_2	357@100<br>297@200<br>229@500<br>168@1000<br>102@2000	500	100	286	80	[39]
Nanotube	Sb/Sb_2S_3 @Carbon hollow tube	608@50<br>556@100<br>446@200<br>349@500<br>263@1000<br>173@2000	3500	1000	200	76	[40]
Nanorod	Sb_2S_3@rGO@NC	513@50<br>353@100<br>253@200<br>147@500<br>76@1000	200	200	89	35	[41]
Microrods	Sb_2S_3-Bi_2S_3@C@rGO	558@100<br>503@200<br>446@300<br>395@400<br>319@500<br>182@1000	80	200	300	60	[42]

Nanoparticle	Sb_2S_3@ MXene	434@50<br>372@100<br>326@200<br>294@500<br>207@1000<br>119@2000	100	50	422	97	[43]
Nanosphere	Sb_2S_3@ CNT	446@50<br>254@100<br>222@500<br>166@1000	50	500	212	95	[44]
Yolk-shell	Sb_2S_3@ NSC	629@50<br>579@100<br>547@200<br>495@500<br>423@1000<br>313@2000<br>210@4000	2000	1000	248	59	[45]
Nanocrystals	CoS_2/Sb_2S_3@NC /CNT	538@100<br>389@200<br>319@500<br>264@1000<br>226@2000<br>133@5000	50	200	453	-	[46]
Nanoparticle	Sb_2S_3@ S, N-doped graphene	547@20<br>535@50<br>500@100<br>460@200<br>420@500<br>340@1000	100	50	500	94	[47]
Nanosheet	Sb_2S_3@ C	500@50<br>450@100<br>400@200<br>250@300<br>175@500<br>100@1000	50	50	350	70	[21]
Nanorod	Bi_2S_3@rGO	579@100<br>562@300<br>541@600<br>292@1000<br>162@3000	1000	100	410	71	[48]
Nanospheres	Se-doped Bi_2S_3@ ReS_2	450@100<br>400@200<br>350@500<br>300@1000<br>100@2000<br>50@3000	900	1000	200	67	[49]

Core-shell	Bi_2S_3@S-doped C	450@50<br>430@100<br>400@200<br>354@400<br>328@600<br>297@800<br>268@1000	250	500	160	46	[50]
Nanocluster	Bi_2S_3/MoS_2@N-dopedC	550@100<br>516@200<br>484@500<br>458@1000<br>424@2000<br>381@5000	400	500	412	85	[51]
Nanowire	Bi_2S_3/Bi_2Se_3	604@50<br>477@100<br>372@250<br>302@500<br>255@1000<br>221@1500<br>196@2000<br>89@3500	1000	500	200	66	[52]
Microsphere	Bi_2S_3@rGO	370@50<br>328@80<br>320@100<br>260@300	1200	100	206	64	[53]
Microsphere	Bi_2S_3@rGO	550@200<br>450@500<br>400@1000<br>300@1500<br>250@2000	300	2000	237	95	[54]
Nanorod	Bi_2S_3@rGO	231@100<br>158@200<br>109@500<br>75@1000	150	200	100.8	63	[55]
Nanorod	Bi_2S_3@iodine-doped graphene	453@50<br>373@100<br>304@200<br>241@500<br>169@1000<br>78@2000	200	100	300	81	[56]

References:

1. De Neufville, J. P.; Moss, S. C.; Ovshinsky, S. R., Photostructural Transformations in Amorphous As2se3 and As2s3 Films. *Journal of Non-Crystalline Solids* **1974,** *13* (2), 191-223 %@ 0022-3093.
2. Shpotyuk, O.; Demchenko, P.; Shpotyuk, Y.; Bujňáková, Z.; Baláž, P., Medium-Range Structural Changes in Glassy As2s3 Driven by High-Energy Mechanical Milling. *Journal of Non-Crystalline Solids* **2019,** *505*, 347-353 %@ 0022-3093.
3. Huang, Y.-F.; Yang, Y.-C.; Tuan, H.-Y., Construction of Strongly Coupled Few-Layer Fepse3-Cnt Hybrids for High Performance Potassium-Ion Storage Devices. *Chemical Engineering Journal* **2023,** *451*, 139013 %@ 1385-8947.
4. Chien, P.-W.; Chang, C.-B.; Tuan, H.-Y., High-Entropy Two-Dimensional Metal Phosphorus Trichalcogenides Boost High-Performance Potassium Ion Storage Devices Via Electrochemical Reconstruction. *Energy Storage Materials* **2023**, 102853 %@ 2405-8297.
5. Liu, C.; Fang, Z.; Li, X.; Zhou, J.; Yang, G.; Peng, L.; Guo, X.; Ding, W.; Hou, W., Rational Design of 3d Porous Niobium Carbide Mxene/Rgo Hybrid Aerogels as Promising Anode for Potassium-Ion Batteries with Ultrahigh Rate Capability. *Nano Research* **2023,** *16* (2), 2463-2473 %@ 1998-0124.
6. Li, Z.; Han, M.; Zhang, Y.; Yuan, F.; Fu, Y.; Yu, J., Single-Layered Mos2 Fabricated by Charge-Driven Interlayer Expansion for Superior Lithium/Sodium/Potassium-Ion-Battery Anodes. *Advanced Science* **2023,** *10* (15), 2207234 %@ 2198-3844.
7. Wu, S.; Feng, Y.; Wu, K.; Jiang, W.; Xue, Z.; Xiong, D.; Chen, L.; Feng, Z.; Wen, K.; Li, Z., Mxene Ti3c2 Generated Tio2 Nanoparticles in Situ and Uniformly Embedded in Rgo Sheets as High Stable Anodes for Potassium Ion Batteries. *Journal of Alloys and Compounds* **2023,** *930*, 167414 %@ 0925-8388.
8. Li, J.; Hu, F.; Wei, H.; Hei, J.; Yin, Y.; Liu, G.; Wang, N.; Wei, H., Confining Mos2/C Nanoparticles on Two-Dimensional Graphene Sheets for High Reversible Capacity and Long-Life Potassium Ions Batteries. *Composites Part B: Engineering* **2023,** *250*, 110424 %@ 1359-8368.
9. Huang, Y.-F.; Yang, Y.-C.; Tseng, Y.-Y.; Tuan, H.-Y., Two Dimensional Mnpse3 Layer Stacking Composites with Superior Storage Performance for Alkali Metal-Ion Batteries. *Journal of Colloid and Interface Science* **2023,** *635*, 336-347 %@ 0021-9797.
10. Lei, Z.; Zheng, J.; He, X.; Wang, Y.; Yang, X.; Xiao, F.; Xue, H.; Xiong, P.; Wei, M.; Chen, Q., Defect-Rich Ws 2–Span Nanofibers for Sodium/Potassium-Ion Batteries: Ultralong Lifespans and Wide-Temperature Workability. *Inorganic Chemistry Frontiers* **2023,** *10* (4), 1187-1196.
11. Feng, Y.; Wu, K.; Wu, S.; Guo, Y.; He, M.; Xue, M., Carbon Quantum Dots-Derived Carbon Nanosphere Coating on Ti3c2 Mxene as a Superior Anode for High-Performance Potassium-Ion Batteries. *ACS Applied Materials & Interfaces* **2023,** *15* (2), 3077-3088 %@ 1944-8244.
12. Wang, M.; Qin, B.; Xu, F.; Yang, W.; Liu, Z.; Zhang, Y.; Fan, H., Hetero-Structural and Hetero-Interfacial Engineering of Mxene@ Bi2s3/Mo7s8 Hybrid for Advanced Sodium/Potassium-Ion Batteries. *Journal of Colloid and Interface Science* **2023,** *650*, 446-455 %@ 0021-9797.
13. Zhang, H.; Xiong, D.; Xie, Y.; Wu, K.; Feng, Z.; Wen, K.; Li, Z.; He, M., Co3c/Mxene Composites Wrapped in N-Rich Carbon as Stable-Performance Anodes for Potassium/Sodium-Ion Batteries. *Colloids and Surfaces A: Physicochemical and Engineering Aspects* **2023,** *656*, 130332 %@ 0927-7757.
14. Li, T.; Wang, Y.; Zhou, Q.; Yuan, L.; Qiao, S.; Ma, M.; Liu, Z.; Chong, S., Snte Nanoparticles Physicochemically Encapsulated by Double Carbon as Conversion-Alloying Anode Materials for Superior Potassium-Ion Batteries. *Journal of Materials Science & Technology* **2023,** *158*, 86-95 %@ 1005-0302.
15. Suo, G.; Zhang, J.; Li, R.; Ma, Z.; Cheng, Y.; Ahmed, S. M., Antimony Anchored in Mos2 Nanosheets with N-Doped Carbon Coating to Boost Potassium Storage Performance. *Materials Today Chemistry* **2023,** *27*, 101300 %@ 2468-5194.

16. Zhou, X.; Wang, Z.; Wang, Y.; Du, F.; Li, Y.; Su, Y.; Wang, M.; Ma, M.; Yang, G.; Ding, S., Graphene Supported Fes2 Nanoparticles with Sandwich Structure as a Promising Anode for High-Rate Potassium-Ion Batteries. *Journal of colloid and interface science* **2023,** *636*, 73-82 %@ 0021-9797.
17. Wang, J.; Wang, B.; Lu, B., Nature of Novel 2d Van Der Waals Heterostructures for Superior Potassium Ion Batteries. *Advanced Energy Materials* **2020,** *10* (24), 2000884 %@ 1614-6832.
18. Ma, M.; Zhang, S.; Yao, Y.; Wang, H.; Huang, H.; Xu, R.; Wang, J.; Zhou, X.; Yang, W.; Peng, Z., Heterostructures of 2d Molybdenum Dichalcogenide on 2d Nitrogen-Doped Carbon: Superior Potassium-Ion Storage and Insight into Potassium Storage Mechanism. *Advanced Materials* **2020,** *32* (22), 2000958 %@ 0935-9648.
19. Wang, H.; Niu, J.; Shi, J.; Lv, W.; Wang, H.; van Aken, P. A.; Zhang, Z.; Chen, R.; Huang, W., Facile Preparation of Mos2 Nanocomposites for Efficient Potassium-Ion Batteries by Grinding-Promoted Intercalation Exfoliation. *Small* **2021,** *17* (34), 2102263 %@ 1613-6810.
20. Cao, J.; Sun, Z.; Li, J.; Zhu, Y.; Yuan, Z.; Zhang, Y.; Li, D.; Wang, L.; Han, W., Microbe-Assisted Assembly of Ti3c2t X Mxene on Fungi-Derived Nanoribbon Heterostructures for Ultrastable Sodium and Potassium Ion Storage. *ACS nano* **2021,** *15* (2), 3423-3433 %@ 1936-0851.
21. Liu, Y.; Tai, Z.; Zhang, J.; Pang, W. K.; Zhang, Q.; Feng, H.; Konstantinov, K.; Guo, Z.; Liu, H. K., Boosting Potassium-Ion Batteries by Few-Layered Composite Anodes Prepared Via Solution-Triggered One-Step Shear Exfoliation. *Nature communications* **2018,** *9* (1), 3645 %@ 2041-1723.
22. Xing, L.; Han, K.; Liu, Q.; Liu, Z.; Chu, J.; Zhang, L.; Ma, X.; Bao, Y.; Li, P.; Wang, W., Hierarchical Two-Atom-Layered Wse2/C Ultrathin Crumpled Nanosheets Assemblies: Engineering the Interlayer Spacing Boosts Potassium-Ion Storage. Energy Storage Mater. 36, 309–317 (2021). 2021.
23. Liu, C.; Zhou, J.; Li, X.; Fang, Z.; Sun, R.; Yang, G.; Hou, W., Surface Modification and in Situ Carbon Intercalation of Two-Dimensional Niobium Carbide as Promising Electrode Materials for Potassium-Ion Batteries. *Chemical Engineering Journal* **2022,** *431*, 133838 %@ 1385-8947.
24. Fan, H. N.; Wang, X. Y.; Yu, H. B.; Gu, Q. F.; Chen, S. L.; Liu, Z.; Chen, X. H.; Luo, W. B.; Liu, H. K., Enhanced Potassium Ion Battery by Inducing Interlayer Anionic Ligands in Mos1. 5se0. 5 Nanosheets with Exploration of the Mechanism. *Advanced Energy Materials* **2020,** *10* (21), 1904162 %@ 1614-6832.
25. Li, J.; Rui, B.; Wei, W.; Nie, P.; Chang, L.; Le, Z.; Liu, M.; Wang, H.; Wang, L.; Zhang, X., Nanosheets Assembled Layered Mos2/Mxene as High Performance Anode Materials for Potassium Ion Batteries. *Journal of Power Sources* **2020,** *449*, 227481 %@ 0378-7753.
26. Li, N.; Sun, L.; Wang, K.; Zhang, J.; Liu, X., Anchoring Mose2 Nanosheets on N-Doped Carbon Nanotubes as High Performance Anodes for Potassium-Ion Batteries. *Electrochimica Acta* **2020,** *360*, 136983 %@ 0013-4686.
27. Share, K.; Cohn, A. P.; Carter, R.; Rogers, B.; Pint, C. L., Role of Nitrogen-Doped Graphene for Improved High-Capacity Potassium Ion Battery Anodes. *ACS nano* **2016,** *10* (10), 9738-9744 %@ 1936-0851.
28. Cao, Y.; Chen, H.; Shen, Y.; Chen, M.; Zhang, Y.; Zhang, L.; Wang, Q.; Guo, S.; Yang, H., Sns2 Nanosheets Anchored on Nitrogen and Sulfur Co-Doped Mxene Sheets for High-Performance Potassium-Ion Batteries. *ACS Applied Materials & Interfaces* **2021,** *13* (15), 17668-17676 %@ 1944-8244.
29. Tian, H.; Yu, X.; Shao, H.; Dong, L.; Chen, Y.; Fang, X.; Wang, C.; Han, W.; Wang, G., Unlocking Few-Layered Ternary Chalcogenides for High-Performance Potassium-Ion Storage. *Advanced Energy Materials* **2019,** *9* (29), 1901560 %@ 1614-6832.
30. Qi, Y.; Yang, Y.; Hou, Q.; Zhang, K.; Zhao, H.; Su, H.; Zhou, L.; Liu, X.; Shen, C.; Xie, K., Uniform-Dispersed Zns Quantum Dots Loading on Graphene as a Promising Anode for Potassium-Ion Batteries. *Chinese Chemical Letters* **2021,** *32* (3), 1117-1120 %@ 1001-8417.
31. Guo, X.; Gao, H.; Wang, S.; Yang, G.; Zhang, X.; Zhang, J.; Liu, H.; Wang, G., Mxene-Based Aerogel Anchored with Antimony Single Atoms and Quantum Dots for High-Performance Potassium-Ion Batteries. *Nano Letters* **2022,** *22* (3), 1225-1232 %@ 1530-6984.

32. Wu, X.; Wang, H.; Zhao, Z.; Huang, B., Interstratification-Assembled 2d Black Phosphorene and V 2 Ct X Mxene as Superior Anodes for Boosting Potassium-Ion Storage. *Journal of Materials Chemistry A* **2020,** *8* (25), 12705-12715.
33. Chang, C.-B.; Chen, K.-T.; Tuan, H.-Y., Large-Scale Synthesis of Few-Layered Copper Antimony Sulfide Nanosheets as Electrode Materials for High-Rate Potassium-Ion Storage. *Journal of Colloid and Interface Science* **2022,** *608*, 984-994 %@ 0021-9797.
34. Zhao, X.; Zhang, C.; Yang, G.; Wu, Y.; Fu, Q.; Zhao, H.; Lei, Y., Bismuth Selenide Nanosheets Confined in Thin Carbon Layers as Anode Materials for Advanced Potassium-Ion Batteries. *Inorganic Chemistry Frontiers* **2021,** *8* (18), 4267-4275.
35. Oh, H. G.; Yang, S. H.; Kang, Y. C.; Park, S. K., N-Doped Carbon-Coated Cose2 Nanocrystals Anchored on Two-Dimensional Mxene Nanosheets for Efficient Electrochemical Sodium-and Potassium-Ion Storage. *International Journal of Energy Research* **2021,** *45* (12), 17738-17748 %@ 0363-907X.
36. Wang, H.; Jia, B.; Zhao, Z.; Luo, C.; Wu, X., Interlayer Spacing Enlarged 2d 1t-Mos2 and V2ctx Mxene as Superior Anodes for Boosting Potassium-Ion Diffusion Coefficient. *Journal of Colloid and Interface Science* **2022,** *618*, 56-67 %@ 0021-9797.
37. Xie, X.-C.; Shuai, H.-L.; Wu, X.; Huang, K.-J.; Wang, L.-N.; Wang, R.-M.; Chen, Y., Engineering Ultra-Enlarged Interlayer Carbon-Containing Vanadium Disulfide Composite for High-Performance Sodium and Potassium Ion Storage. *Journal of Alloys and Compounds* **2020,** *847*, 156288 %@ 0925-8388.
38. Liu, H.; He, Y.; Cao, K.; Wang, S.; Jiang, Y.; Liu, X.; Huang, K. J.; Jing, Q. S.; Jiao, L., Stimulating the Reversibility of Sb2s3 Anode for High-Performance Potassium-Ion Batteries. *Small* **2021,** *17* (10), 2008133 %@ 1613-6810.
39. Wang, T.; Shen, D.; Liu, H.; Chen, H.; Liu, Q.; Lu, B., A Sb2s3 Nanoflower/Mxene Composite as an Anode for Potassium-Ion Batteries. *ACS Applied Materials & Interfaces* **2020,** *12* (52), 57907-57915 %@ 1944-8244.
40. Wu, Y.; Zheng, J.; Tong, Y.; Liu, X.; Sun, Y.; Niu, L.; Li, H., Carbon Hollow Tube-Confined Sb/Sb2s3 Nanorod Fragments as Highly Stable Anodes for Potassium-Ion Batteries. *ACS Applied Materials & Interfaces* **2021,** *13* (43), 51066-51077 %@ 1944-8244.
41. Chong, S.; Qiao, S.; Wei, X.; Li, T.; Yuan, L.; Dong, S.; Huang, W., Sb2s3-Based Conversion-Alloying Dual Mechanism Anode for Potassium-Ion Batteries. *Iscience* **2021,** *24* (12 %@ 2589-0042).
42. Li, K.; Liu, X.; Qin, Y.; Zhao, Z.; Xu, Y.; Yi, Y.; Guan, H.; Fu, Y.; Liu, P.; Li, D., Sb2s3-Bi2s3 Microrods with the Combined Action of Carbon Encapsulation and Rgo Confinement for Improving High Cycle Stability in Sodium/Potassium Storage. *Chemical Engineering Journal* **2021,** *414*, 128787 %@ 1385-8947.
43. Zhang, P.; Zhu, Q.; Wei, Y.; Xu, B., Achieving Stable and Fast Potassium Storage of Sb2s3@ Mxene Anode Via Interfacial Bonding and Electrolyte Chemistry. *Chemical Engineering Journal* **2023,** *451*, 138891 %@ 1385-8947.
44. Li, M.; Huang, F.; Pan, J.; Li, L.; Zhang, Y.; Yao, Q.; Zhou, H.; Deng, J., Amorphous Sb2s3 Nanospheres in-Situ Grown on Carbon Nanotubes: Anodes for Nibs and Kibs. *Nanomaterials* **2019,** *9* (9), 1323 %@ 2079-4991.
45. Xiao, B.; Zhang, H.; Sun, Z.; Li, M.; Fan, Y.; Lin, H.; Liu, H.; Jiang, B.; Shen, Y.; Wang, M.-S., Achieving High-Capacity and Long-Life K+ Storage Enabled by Constructing Yolk-Shell Sb2s3@ N, S-Doped Carbon Nanorod Anodes. *Journal of Energy Chemistry* **2023,** *76*, 547-556 %@ 2095-4956.
46. Li, X.; Liang, H.; Liu, X.; Sun, R.; Qin, Z.; Fan, H.; Zhang, Y., Ion-Exchange Strategy of Cos2/Sb2s3 Hetero-Structured Nanocrystals Encapsulated into 3d Interpenetrating Dual-Carbon Framework for High-Performance Na+/K+ Batteries. *Chemical Engineering Journal* **2021,** *425*, 130657 %@ 1385-8947.
47. Lu, Y.; Chen, J., Robust Self-Supported Anode by Integrating Sb 2 S 3 Nanoparticles with S, N-Codoped Graphene to Enhance K-Storage Performance. *Science China Chemistry* **2017,** *60*, 1533-1539 %@ 1674-7291.

48. Nithya, C.; Modigunta, J. K. R.; In, I.; Kim, S.; Gopukumar, S., Bi2s3 Nanorods Deposited on Reduced Graphene Oxide for Potassium-Ion Batteries. *ACS Applied Nano Materials* **2023,** *6* (7), 6121-6132 %@ 2574-0970.

49. Lin, J.; Lu, S.; Zhang, Y.; Zeng, L.; Zhang, Y.; Fan, H., Selenide-Doped Bismuth Sulfides (Bi2s3-Xsex) and Their Hierarchical Heterostructure with Res2 for Sodium/Potassium-Ion Batteries. *Journal of Colloid and Interface Science* **2023,** *645*, 654-662 %@ 0021-9797.

50. Wang, C.; Lu, J.; Tong, H.; Wu, S.; Wang, D.; Liu, B.; Cheng, L.; Lin, Z.; Hu, L.; Wang, H., Structural Engineering of Sulfur-Doped Carbon Encapsulated Bismuth Sulfide Core-Shell Structure for Enhanced Potassium Storage Performance. *Nano Research* **2021,** *14* (10), 3545-3551 %@ 1998-0124.

51. Qin, Y.; Zhang, Y.; Wang, J.; Zhang, J.; Zhai, Y.; Wang, H.; Li, D., Heterogeneous Structured Bi2s3/Mos2@ Nc Nanoclusters: Exploring the Superior Rate Performance in Sodium/Potassium Ion Batteries. *ACS Applied Materials & Interfaces* **2020,** *12* (38), 42902-42910 %@ 1944-8244.

52. Hsieh, Y.-Y.; Tuan, H.-Y., Architectural Van Der Waals Bi2s3/Bi2se3 Topological Heterostructure as a Superior Potassium-Ion Storage Material. *Energy Storage Materials* **2022,** *51*, 789-805 %@ 2405-8297.

53. Sun, X.; Wang, L.; Li, C.; Wang, D.; Sikandar, I.; Man, R.; Tian, F.; Qian, Y.; Xu, L., Dandelion-Like Bi 2 S 3/Rgo Hierarchical Microspheres as High-Performance Anodes for Potassium-Ion and Half/Full Sodium-Ion Batteries. *Nano Research* **2021**, 1-8 %@ 1998-0124.

54. Liu, Y.; Li, M.; Zheng, Y.; Lin, H.; Wang, Z.; Xin, W.; Wang, C.; Du, F., Boosting Potassium-Storage Performance Via the Functional Design of a Heterostructured Bi 2 S 3@ Rgo Composite. *Nanoscale* **2020,** *12* (48), 24394-24402.

55. Yuan, L.; Zhou, Q.; Li, T.; Wang, Y.; Liu, Z.; Chong, S., Promoting Superior K-Ion Storage of Bi2s3 Nanorod Anode Via Graphene Physicochemical Protection and Electrolyte Stabilization Effect. *Applied Energy* **2022,** *322*, 119471 %@ 0306-2619.

56. Wei, Y.; Hou, W.; Zhang, P.; Soomro, R. A.; Xu, B., Bi2s3 Nanorods Encapsulated in Iodine-Doped Graphene Frameworks with Enhanced Potassium Storage Properties. *Chinese Chemical Letters* **2022,** *33* (6), 3212-3216 %@ 1001-8417.
